# The effectiveness and safety of acupuncture combined with medication in the treatment of perimenopausal insomnia: a systematic review and meta-analysis

**DOI:** 10.3389/fneur.2025.1476719

**Published:** 2025-03-13

**Authors:** Shengwen Jiang, Yuan Zhang, Yingzhe Sun

**Affiliations:** ^1^School of Graduate, Heilongjiang University of Chinese Medicine, Harbin, China; ^2^Department of Acupuncture II, The Second Affiliated Hospital of Heilongjiang University of Chinese Medicine, Harbin, China

**Keywords:** acupuncture, medication, combination therapy, perimenopause, menopause, insomnia, systematic review, meta-analysis

## Abstract

**Introduction:**

The aim of this study is to evaluate the effectiveness and safety of the combination therapy of acupuncture and medication in the treatment of perimenopausal insomnia (PMI). This research seeks to provide scientific evidence for clinical practice, optimize treatment protocols, and enhance the sleep quality and overall quality of life for women experiencing perimenopausal insomnia.

**Methods and analysis:**

A comprehensive search was conducted across 8 databases, including the China National Knowledge Infrastructure (CNKI), Wanfang Academic Journal Full-text Database (Wanfang), Chongqing VIP Database (CQVIP), China Biology Medicine Disc (CBM), PubMed, Web of Science, Excerpta Medica Database (EMBASE), and Cochrane Library, from their establishment to July 1, 2024. Outcome measures were analyzed using Review Manager 5.4 and Stata 15.0 software. The included randomized controlled trials (RCTs) involved 1,187 patients with perimenopausal sleep disorders (596 in the experimental group and 591 in the control group). The analysis indicated that compared to Western medication alone, the combination therapy showed better efficacy [risk ratio (RR) = 1.24, 95% confidence interval (CI) (1.17, 1.31), *p* < 0.00001] and safety [RR = 0.31, 95%CI (0.18, 0.53), *p* < 0.0001]. It also demonstrated more significant improvements in Pittsburgh Sleep Quality Index (PSQI) [mean difference (MD) = −2.77, 95%CI (−4.11, −1.43), *p* < 0.0001], Hamilton Anxiety Rating Scale (HAMA) scores [MD = −3.45, 95%CI (−3.94, −2.97), *p* < 0.00001], Kupperman Menopausal Index (KMI) [MD = −1.46, 95%CI (−2.23, −0.70), *p =* 0.0002], Traditional Chinese Medicine Syndromes (TCMS) scores [MD = −2.45, 95%CI (−3.85, −1.04), *p* = 0.0006], and hormone levels, including Luteinizing Hormone (LH) [MD = −4.17, 95%CI (−7.42, −0.93), *p* = 0.01], Follicle-Stimulating Hormone (FSH) [MD = −10.50, 95%CI (−14.80, −6.20), *p* < 0.00001], and Estradiol (E_2_) [MD = 12.15, 95%CI (6.79, 17.51), *p* < 0.00001].

**Discussion:**

The combination therapy demonstrates great efficacy and safety for PMI patients, representing an innovative integrative alternative treatment with high clinical application value.

**Systematic review registration:**

https://www.crd.york.ac.uk/PROSPERO/view/CRD42024564357, PROSPERO CRD42024564357.

## 1 Introduction

Before entering menopause, women undergo perimenopause—a critical transitional phase characterized by significant reproductive and hormonal changes (1). According to the STRAW staging criteria, perimenopause includes the early menopausal transition stage (−2) and the late menopausal transition stage (−1), marking the transition from the reproductive period to menopause. During this period, ovarian reserve gradually decreases, menstrual cycles become irregular, and hormone levels experience significant fluctuations (1). Perimenopausal syndrome (PMS), also known as menopausal syndrome (MPS), refers to a series of signs and psychological symptoms caused by women before and after menopause, such as palpitation, hot flashes, night sweats, vaginal dryness, breast pain, sleep disorders, which are rooted in the fluctuation and reduction of sex hormones (2, 3). Insomnia, as one of the common complaints of perimenopausal women, seriously threatens the quality of life of women around the world. In recent years, the prevalence of PMI has attracted increasing attention. Although developed countries have made significant progress in managing perimenopausal symptoms, developing countries still face high prevalence and limited treatment options (4, 5). In China, PMI affects a considerable proportion of the female population, many of whom experience moderate to severe symptoms, which not only interferes with daily activities and reduces the quality of life, but also leads to other health problems, such as depression, anxiety and cardiovascular disease (4, 6).

Currently, the main treatments for PMI include hormone replacement therapy (HRT) and non-hormonal therapies (7). HRT, one of the treatments for severe perimenopausal symptoms, may pose risks such as increased chances of breast cancer, thromboembolic events, and cardiovascular issues (8–10). Non-hormonal therapies, such as sleeping medications, have significant side effects and withdrawal reactions, making the quest for effective and safer alternative treatments crucial for improving PMI (11). Studies have shown that traditional Chinese treatments such as acupuncture and herbal medication are efficacious in improving PMI symptoms (12, 13). Therefore, another promising alternative method, acupuncture combined with medication, has been gradually explored by researchers. This method has shown the effect of relieving perimenopausal symptoms with fewer side effects (14, 15). The combination of acupuncture and medication, either Traditional Chinese Medication (TCM) or Western Medication (WM), can regulate neuroendocrine function, improve sleep quality, reduce levels of anxiety and depression, making it a well-supported option in treating PMI (14). This article aims to explore the effectiveness and safety of the combination therapy of acupuncture and medication in the treatment of PMI through a systematic review and meta-analysis, providing insights into its potential benefits and mechanisms of action.

## 2 Methods and analysis

### 2.1 Study registration

According to the guidelines (16), we registered the systematic review protocol in PROSPERO on July 13, 2024 (Registration Number: CRD42024564357).

### 2.2 Eligibility criteria

#### 2.2.1 Study designs

Only published RCTs were considered for inclusion.

#### 2.2.2 Participants

Only peri-menopausal women who met the diagnostic criteria of insomnia were considered for inclusion, with no restrictions on age, duration of the condition, ethnicity, country, or educational level. For specific details, refer to Table 1 and the accompanying note.

**Table 1:** Information supplement.

Study	Diagnostic criteria (P/I)	Duration	Outcome measures
Kang (18)	⑧	4w	Clinical Effective Rate, PSQI, LH, FSH, E_2_, HAMA
Lu et al. (19)	①⑦	4w	Clinical Effective Rate, PSQI, FSH, E_2_
Quan and Yan (20)	②⑫	1 m	Clinical Effective Rate, PSQI, LH, FSH, E_2_
Sun et al. (21)	⑪	2 m	Clinical Effective Rate, PSQI, LH, FSH, E_2_
Xu (22)	①⑥	4w	Clinical Effective Rate, PSQI, KMI, TCMS
Xu and Zhao (23)	-	30d	Clinical Effective Rate, PSQI, LH, FSH, E_2_, KMI, AEIR
Xu (24)	-	-	Clinical Effective Rate, PSQI, LH, FSH, E_2_, AEIR
Yan et al. (25)	⑨	16w	Clinical Effective Rate, PSQI, LH, FSH, E_2_, HAMA, AEIR, TCMS
Zhang and Zhou (26)	②⑫	4w	Clinical Effective Rate, PSQI, LH, FSH, E_2_, HAMA, AEIR
Ran and Wang (27)	③⑫	4w	PSQI, LH, FSH, E_2_, AEIR
Zeng (28)	⑦⑬	3w	Clinical Effective Rate, PSQI, TCMS
Xue et al. (29)	④⑦	4w	Clinical Effective Rate, PSQI, LH, FSH, E_2_, HAMA, KMI, AEIR
Zhou (30)	⑤⑩	4w	Clinical Effective Rate, PSQI, FSH, E_2_, KMI, TCMS
Zhu et al. (31)	⑦	4w	PSQI

① Obstetrics and Gynecology ② Obstetrics and Gynecology, 2008:14. ③ Chinese Obstetrics and Gynecology, 3rd ed, 2014:2537. ④ Obstetrics and Gynecology, 6th ed, 2004:9. ⑤ Obstetrics and Gynecology, 2018:365. ⑥ Classification and Diagnostic Criteria of Mental Disorders in China. ⑦ China Classification and Diagnostic Criteria of Mental Disorders, 3rd ed, 2001:118–119. ⑧ Key Changes in the 5th Edition of the Diagnostic and Statistical Manual of Mental Disorders (DSM-5), 2013, 23(4):289–290. ⑨ Diagnostic and statistical mannual of mental disorders, 4th ed, text version (DSM-IV), 2000. ⑩ Diagnosing the Diagnostic and Statistical Manual of Mental Disorders: Fifth Edition, 2018:193. ⑪ Guidelines for the Diagnosis and Treatment of Insomnia in China, 2017, 97(24):1844–1856. ⑫ Guidelines for the Diagnosis and Treatment of Insomnia in Adults in China (2017 Edition), 2018, 51(5):324–335. ⑬ Clinical Diagnosis and Treatment Guidelines: Obstetrics and Gynecology Volume, 2009.

#### 2.2.3 Interventions

The treatment group received the combination of acupuncture and medication (both TCM and WM are acceptable), while the control group was treated with WM (Estazolam).

#### 2.2.4 Outcome measures

Primary outcome measures: Clinical Effective Rate, Adverse Event Incidence Rate (AEIR).

Secondary outcome measures: PSQI, LH, FSH, E_2_, HAMA scores, KMI, TCMS scores.

#### 2.2.5 Language

Only articles reported in Chinese or English were included.

### 2.3 Search strategy

Searches were conducted in the databases of PubMed, Excerpta Medica Database (EMBASE), Cochrane Library, Web of Science, China Biology Medicine Disc (CBM), China National Knowledge Infrastructure (CNKI), Wanfang Academic Journal Full-text Database (Wanfang), and Chongqing VIP Database (CQVIP). The search, which was conducted up to July 1, 2024, utilized both MeSH terms and text word. The search terms include “acupuncture,” “combination therapy,” “perimenopause,” “insomnia,” “RCTs,” etc. Taking PubMed search strategy as an example, refer to Table 2.

**Table 2:** Search strategy (PubMed).

Search	Query
#1	Perimenopause [MeSH Terms]
#2	Perimenopausal Syndrome [Text Word] OR Menopausal Transition [Text Word]
#3	#1 OR #2
#4	Insomnia [MeSH Terms]
#5	Sleep Difficulty [Text Word] OR Difficulty Falling Asleep [Text Word] OR Sleep Disorder [Text Word] OR Poor Sleep Quality [Text Word] OR Sleep Deprivation [Text Word] OR Inability Closing Eyes [Text Word] OR Inability Lying Down [Text Word] OR Sleeplessness [Text Word]
#6	#4 OR #5
#7	Randomized Controlled Trial [MeSH Terms]
#8	Random [Text Word] OR Control [Text Word] OR Intervention [Text Word] OR Randomized Controlled [Text Word] OR Randomized Controlled Study [Text Word] OR Clinical Trial [Text Word] OR Clinical Study [Text Word] OR Efficacy Observation [Text Word] OR Efficacy Evaluation [Text Word]
#9	#7 OR #8
#10	Acupuncture [MeSH Terms]
#11	Acupuncture [Text Word] AND Moxibustion [Text Word] OR Scalp Acupuncture [Text Word] OR Neck Acupuncture [Text Word] OR Auricular Acupuncture [Text Word] OR Tongue Acupuncture [Text Word] OR Hand Acupuncture [Text Word] OR Foot Acupuncture [Text Word] OR Abdominal Acupuncture [Text Word] OR Body Acupuncture [Text Word] OR Intradermal Needle [Text Word] OR Electroacupuncture [Text Word] OR Warm Acupuncture [Text Word] OR Fire Needle [Text Word] OR Press Needle [Text Word] OR Thread Embedding [Text Word] OR Needle Embedding [Text Word] OR Acupoint Catgut Embedding [Text Word]
#12	#10 OR #11
#13	#3 AND #6 AND #9 AND #12

### 2.4 Study selection

Two reviewers (SJ and YZ) independently screened the literature using Endnote 20 software. Studies that failed to meet the selection criteria were excluded. Those with uncertain eligibility underwent a detailed review to ascertain their suitability. Any discrepancies were resolved by consulting a third reviewer (YS).

### 2.5 Data organisation

Initially, data extraction was performed, with two reviewers (SJ and YZ) independently extracting and cross-checking the data. Any discrepancies were resolved by consulting a third reviewer (YS) who participated in the discussion and decision-making process. The data extraction encompasses the first author, publication date, interventions, sample size, age, course, acupuncture points, medication dosages, diagnostic criteria, duration, and outcome measures.

### 2.6 Quality assessment

The quality assessment of the included studies and the evaluation of risk of bias were conducted in accordance with the guidelines provided in the Cochrane Handbook for Systematic Reviews of Interventions (17). This includes seven items: (1) Generation of a random sequence (selection bias); (2) Concealment of allocation (selection bias); (3) Participant and personnel blinding (performance bias); (4) Outcome assessment blinding (detection bias); (5) Management of incomplete outcome data (attrition bias); (6) Selective data reporting (reporting bias); (7) Other potential biases (other bias). Each item was evaluated as having a “low,” “unclear,” or “high” risk of bias. In case of discrepancies, a third reviewer (SYZ) was consulted to reach a consensus.

### 2.7 Missing data handling

For missing data, we will prioritize contacting the original authors to obtain the missing information. If the data cannot be supplemented, we will use sensitivity analysis to explore the potential impact of missing data on the final effect evaluation and assess different data handling strategies (such as imputation or exclusion).

### 2.8 Data synthesis

Meta-analysis was performed using Review Manager 5.4 and Stata 15.0 software. The RR was used for dichotomous variables (count data), and the MD was applied for continuous variables (measurement data). The CI for all effect sizes were set at 95%.

### 2.9 Heterogeneity assessment

Heterogeneity was assessed using the *I^2^* statistic and *p*-value. When *I^2^* ≤ 50% and *p* > 0.1, it indicates low heterogeneity, and a fixed-effect model is applied. When *I^2^* > 50% and *p* < 0.1, it suggests substantial heterogeneity, and a random-effects model is applied. If necessary, subgroup analysis, sensitivity analysis, and regression analysis are performed.

## 3 Results

### 3.1 Study selection

**Figure 1:**
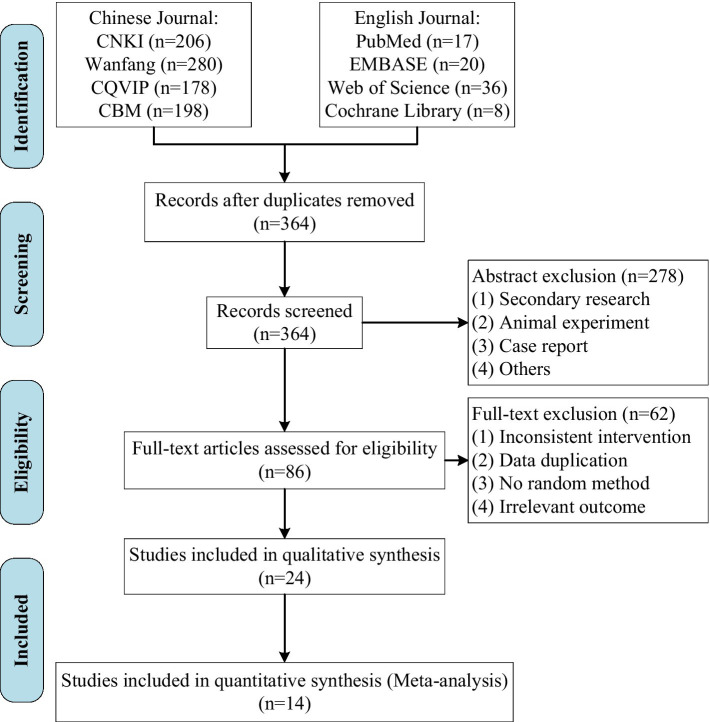
Study flow diagram.

A total of 944 relevant studies were initially retrieved. After applying various inclusion criteria and exclusions, 14 studies (18–31) were ultimately included, as detailed in Figure 1.

### 3.2 Basic characteristics of the studies

A total of 14 RCTs (18–31) on acupuncture combined with medication for the treatment of PMI included 1,187 patients (596 in the experimental group and 591 in the control group), with baseline data being largely similar. Among these, 12 studies (18–26, 28–30) reported clinical effective rate, 6 studies (23–27, 29) reported the incidence of adverse events, 14 studies (18–31) reported PSQI, 9 studies (18, 20, 21, 23–27, 29) reported LH levels, 11 studies (18–21, 23–27, 29, 30) reported FSH levels, 11 studies (18–21, 23–27, 29, 30) reported E_2_ levels, 4 studies (18, 25, 26, 29) reported HAMA scores, 4 studies (22, 23, 29, 30) reported KMI, and 4 studies (22, 25, 28, 30) reported TCMS scores. Regarding interventions, 11 studies (18–28) compared acupuncture combined with TCM against sole use of the WM (Estazolam), while 3 studies (29–31) compared acupuncture combined with WM (Estazolam) against sole use of the WM (Estazolam), as detailed in Tables 1, 3.

**Table 3:** Basic characteristics of the studies.

Study ID	Experimental treatment	Control treatment	Sample size (E/C)	Age [mean (SD)] (E/C)	Course [mean (SD)] (E/C)	Acupuncture points	Medication dosages (per dose)
Kang (18)	Acupuncture and Xiang Fu Tang	Estazolam	43/43	50.45 (3.92)y/49.30 (3.15)y	2.91 (0.56)y/3.11 (0.73)y	BL_62_, KI_6_, SP_6_, BL_20_, ST_36_	1–2 mg
Lu et al. (19)	Acupuncture and Bai Zi Yang Xin Tang	Estazolam	46/45	46.13 (−)y/46.13 (−)y	5.1 (0.7)y/4.6 (0.8)y	DU_20_, EX-HN_1_, BL_15_, BL_23_, ST_25_, RN_4_, EX-CA_1_, SP_6_	2 mg
Quan and Yan (20)	Acupuncture and Gan Mai Da Zao Tang	Estazolam	47/48	49.76 (2.71)y49.14 (2.93)y	2.25 (0.78)y/2.12 (0.67)y	DU_20_, EX-HN_1_, HT_5_, HT_7_, SP_6_, KI_3_, KI_4_, BL_62_, KI_6_	1–2 mg
Sun et al. (21)	Acupuncture and Qing Re An Shen Tang	Estazolam	53/53	51.62 (3.11)y/51.66 (3.12)y	27.82 (1.21)m/27.84 (1.23)m	DU_20_, EX-HN_3_, GB_20_, KI_6_, HT_7_, SP_6_, BL_15_, AnMian, PC_6_	2 mg
Xu (22)	Acupuncture and Wen Dan Tang	Estazolam	38/39	50.81 (3.04)y/50.18 (3.05)y	11.13 (4.85)m/11.52 (4.74)m	DU_20_, EX-HN_1_, DU_24_, AnMian, HT_7_, PC_6_, ST_40_, SP_6_	1 mg
Xu and Zhao (23)	Acupuncture and Wen An Shen Yang Xue Tang	Estazolam	53/53	51.01 (5.22)y/51.24 (5.33)y	11.89 (2.11)m/12.05 (2.16)m	1.5 cun anterior, posterior, left, and right to DU_20_, 0.5 cun above EX-HN_3_, 0.5 cun above GB_14_ on the left and right, DU_24_, Bilateral GB_13_	1 mg
Xu (24)	Acupuncture and Suan Zao Ren Tang	Estazolam	31/31	51.82 (3.94)y/50.88 (4.88)y	8.24 (3.76)m/7.58 (3.42)m	HT_7_, RN_4_, AnMian, DU_20_, PC_6_, EX-HN_5_, RN_12_	-
Yan et al. (25)	Acupuncture and Xiang Fu Tang	Estazolam	59/57	50.8 (7.6)y/49.6 (7.2)y	3.1 (0.5)y/2.9 (0.4)y	EX-HN_1_, AnMian, HT_7_, SP_6_, BL_18_, BL_13_, GB_20_, ST_36_	1 mg
Zhang and Zhou (26)	Acupuncture and Bai He Di Huang Tang	Estazolam	39/39	52.76 (2.81)y/52.14 (2.63)y	2.75 (0.77)y/2.09 (0.64)y	DU_20_, HT_7_, DU_24_, EX-HN_1_, GB_13_, PC_6_, SP_6_	1-2 mg
Ran and Wang (27)	Acupuncture and Suan Zao Ren Tang	Estazolam	43/43	50.63 (7.59)y/50.51 (7.57)y	10.72 (1.60)m/10.52 (1.57)m	DU_20_, HT_7_, AnMian, EX-HN_5_, RN_4_, PC_6_, RN_12_, RN_10_	-
Zeng (28)	Acupuncture and Tiao Jing An Shen Tang	Estazolam	30/30	50.70 (3.12)y/50.97 (3.17)y	11.02 (3.19)m/10.63 (2.92)m	the lower 2/5 of the vasomotor area, the upper 1/5 of the sensory area, GB_4_-GB_6_, GB_9_-SJ_20_, DU_24_-DU_20_	1 mg
Xue et al. (29)	Acupuncture and Estazolam	Estazolam	42/41	48.35 (2.37)y/47.75 (3.10)y	7.34 (1.63)m/8.02 (1.46)m	EX-HN_1_, AnMian, DU_20_, BL_62_, LI_4_, ST_40_, LR_14_, LR_2_, LR_3_, BL_18_, KI_6_, SP_6_, ST_36_	2 mg
Zhou (30)	Acupuncture and Estazolam	Estazolam	35/32	50.37 (2.47)y/49.71 (2.71)y	7.89 (2.91)m/7.94 (2.96)m	HT_7_, Bilateral auricular points: Heart, Kidney, Sympathetic, Endocrine, Subcortex	1 mg
Zhu et al. (31)	Acupuncture and Estazolam	Estazolam	37/37	49.86 (3.15)y/49.27 (3.58)y	2.99 (4.24)y/2.97 (3.42)y	DU_20_, DU_24_, EX-HN_1_, AnMian, HT_7_, LR_3_, KI_3_, RN_12_, ST_25_, SP_9_	1 mg

E, Experimental Group; C, Control Group; SD, Standard Deviation; P, Perimenopause; I, Insomnia; w, week(s); m, month(s); y, year(s); AEIR, Adverse Events Incidence Rate; PSQI, Pittsburgh Sleep Quality Index; LH, Luteinizing Hormone; FSH, Follicle-Stimulating Hormone; E2, Estradiol; HAMA, Hamilton Anxiety Scale; KMI, Kupperman Menopausal Index; TCMS, Traditional Chinese Medicine Syndromes.

### 3.3 Risk of bias assessment

**Figure 2:**
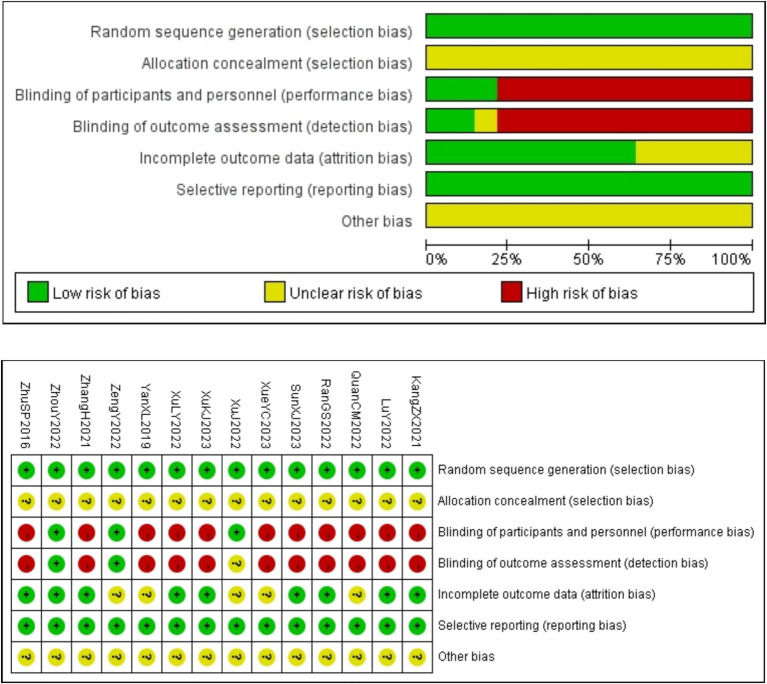
Risk of bias assessment.

All 14 RCTs (18–31) employed random methods (10 studies (18, 20, 21, 23, 25–27, 29–31) used a random number table method, 2 studies (22, 28) used simple randomization, 1 study (24) used computer-generated randomization, and 1 study (19) used other random allocation schemes), ensuring proper randomization and thereby minimizing selection bias. However, none of the studies reported allocation concealment, which raises concerns about potential selection bias, as the allocation process could be inadvertently influenced by researchers or participants. Blinding was implemented in 3 studies (22, 28, 30) for both participants and personnel, and outcome assessment blinding was also reported in these same 3 studies (22, 28, 30), although the reporting in 1 study (22) was not entirely clear. Due to the specific nature of acupuncture procedures, it is practically impossible to implement blinding. However, since 3 studies (22, 28, 30) explicitly mentioned the use of blinding, we assessed them as low risk. The lack of blinding in the remaining studies may introduce performance and detection biases, potentially affecting the objectivity of the results. Additionally, 5 studies (20, 22, 25, 28, 29) had issues with missing outcome data. For the missing data, we will take appropriate measures for handling it in the subsequent analysis. No selective reporting bias was observed across the assessments, and no other types of bias were identified. Further details are provided in Figure 2.

### 3.4 Primary outcome measures

#### 3.4.1 Clinical effective rate

**Figure 3:**
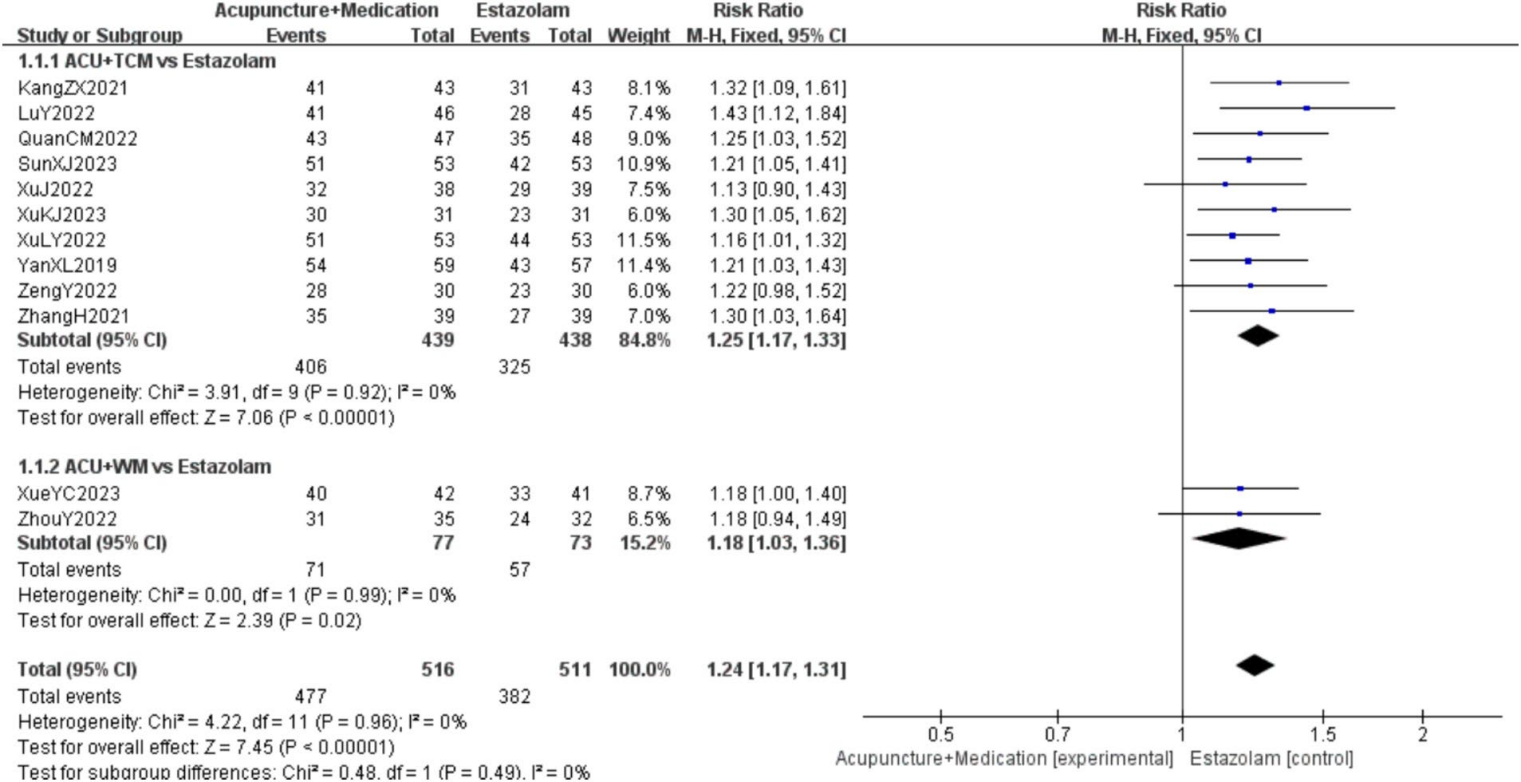
Clinical effective rate forest plot.

A total of 1,027 patients in 12 studies (18–26, 28–30) reported the clinical effective rate of acupuncture combined with medication in treating PMI. Analysis of the extracted data revealed low heterogeneity (*p* = 0.96 > 0.1; *I^2^* = 0%), so a fixed-effect model was selected. The results indicated that, compared to the control group, acupuncture combined with medication significantly improved insomnia symptoms in perimenopausal women [RR = 1.24, 95%CI (1.17, 1.31), *Z* = 7.45, *p* < 0.00001]. Subgroup analysis indicated that the effect of acupuncture combined with TCM [RR = 1.25, 95%CI (1.17, 1.33), *Z* = 7.06, *p* < 0.00001] might be slightly better than that of acupuncture combined with WM [RR = 1.18, 95%CI (1.03, 1.36), *Z* = 2.39, *p* = 0.02 < 0.05], but this advantage did not reach a significant difference due to small sample sizes, as detailed in Figure 3.

#### 3.4.2 AEIR

**Figure 4:**
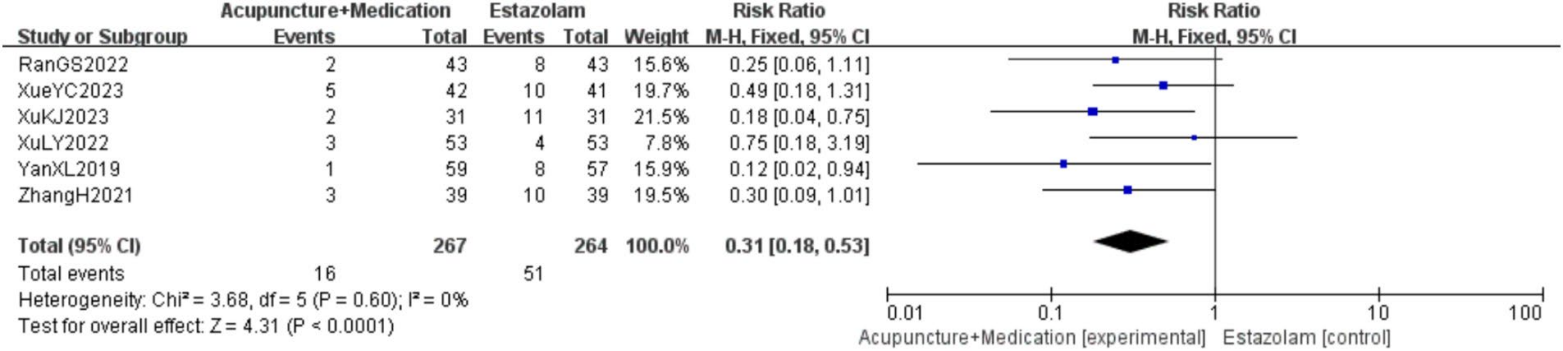
AEIR forest plot.

A total of 531 patients in 6 studies (23–27, 29) reported the AEIR of acupuncture combined with medication in treating PMI. Analysis of the extracted data revealed low heterogeneity (*p* = 0.60 > 0.1; *I^2^* = 0%), so a fixed-effect model was selected. The results indicated that, compared to the control group, the incidence of adverse events of the combination therapy was significantly reduced with statistical significance [RR = 0.31, 95%CI (0.18, 0.53), *Z* = 4.31, *p* < 0.0001], as detailed in Figure 4.

### 3.5 Secondary outcome measures

#### 3.5.1 PSQI

**Figure 5:**
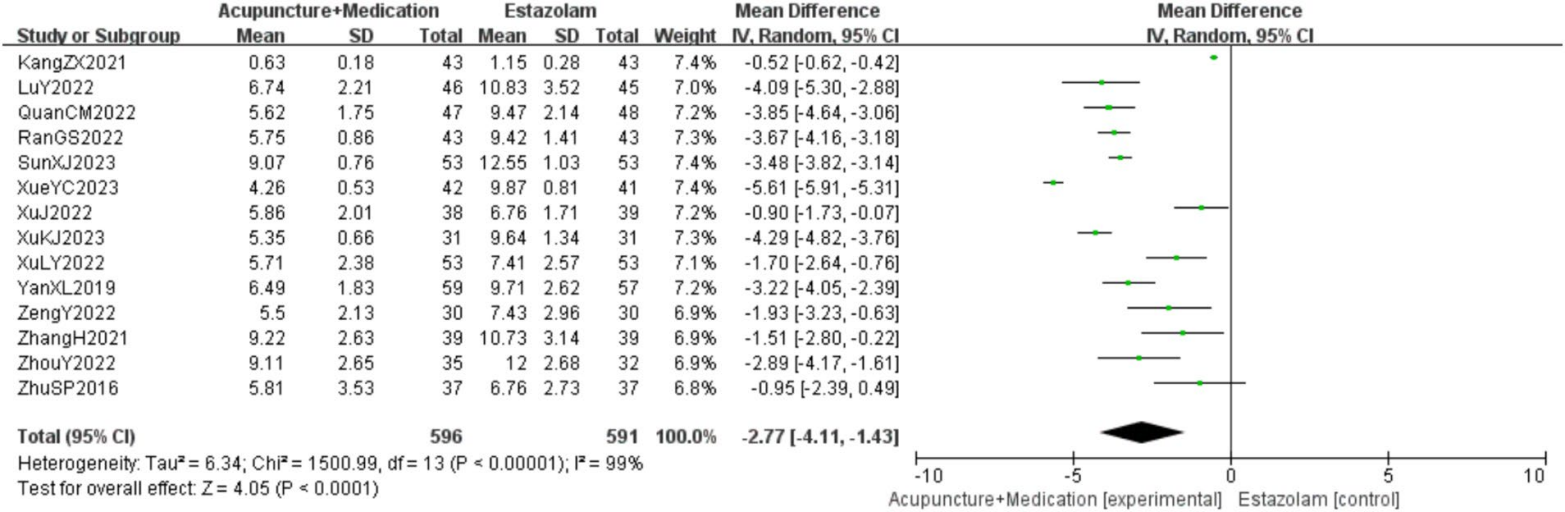
PSQI forest plot.

A total of 1,187 patients in 14 studies (18–31) reported the PSQI of acupuncture combined with medication in treating PMI. Analysis of the extracted data revealed high heterogeneity (*p* < 0.00001; *I^2^* = 99%), so a random-effects model was selected. The results indicated that, compared to the control group, combination therapy was more effective in improving PSQI with statistical significance [MD = −2.77, 95%CI (−4.11, −1.43), *Z* = 4.05, *p* < 0.0001]. Sensitivity analysis, as shown in the Supplementary image, indicated stable results. Systematically removing studies one at a time fails to make heterogeneity reduce significantly. Subgroup analysis was followed by regression analysis, as detailed in Figure 5.

#### 3.5.2 LH

**Figure 6:**
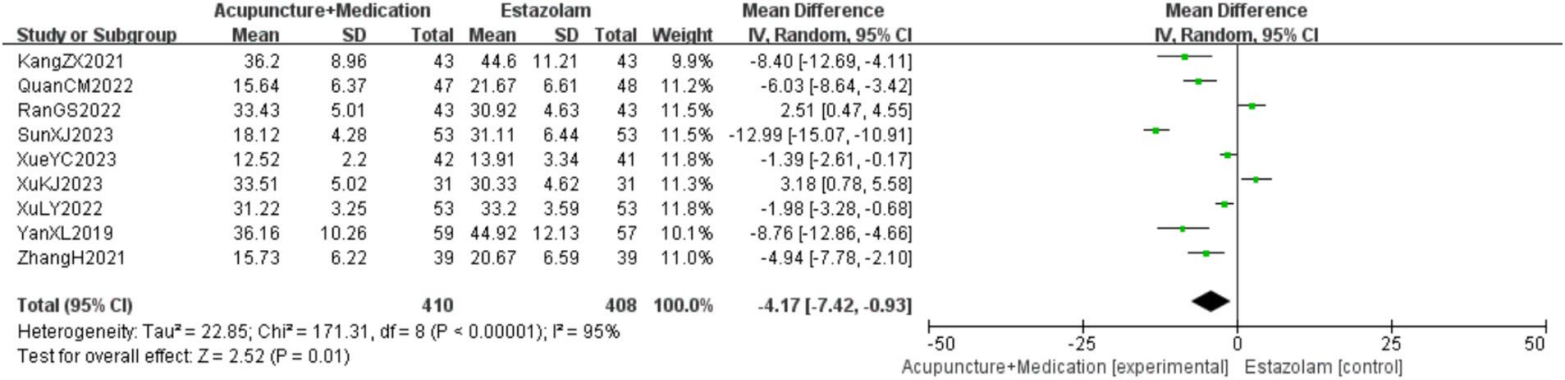
LH forest plot.

A total of 818 patients in 9 studies (18, 20, 21, 23–27, 29) reported the LH levels of acupuncture combined with medication in treating PMI. Analysis of the extracted data revealed high heterogeneity (*p* < 0.00001; *I^2^* = 95%), so a random-effects model was selected. The results indicated that, compared to the control group, combination therapy was more effective in improving LH levels with statistical significance [MD = −4.17, 95%CI (−7.42, −0.93), *Z* = 2.52, *p* = 0.01 < 0.05]. Sensitivity analysis, as shown in the Supplementary image, indicated stable results. Systematically removing studies one at a time fails to make heterogeneity reduce significantly. After subgroup analysis, regression analysis was conducted, as detailed in Figure 6.

#### 3.5.3 FSH

**Figure 7:**
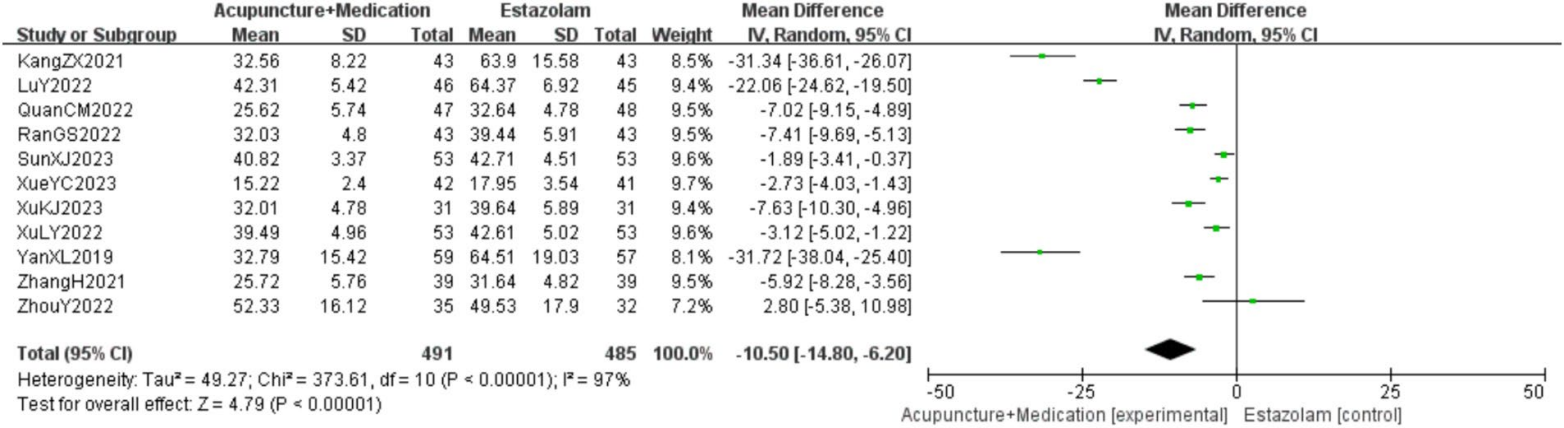
FSH forest plot.

A total of 976 patients in 11 studies (18–21, 23–27, 29, 30) reported the FSH levels of acupuncture combined with medication in treating PMI. Analysis of the extracted data revealed high heterogeneity (*p* < 0.00001; *I^2^* = 97%), so a random-effects model was selected. The results indicated that, compared to the control group, combination therapy was more effective in improving FSH levels with statistical significance [MD = −10.50, 95%CI (−14.80, −6.20), *Z* = 4.79, *p* < 0.00001]. Sensitivity analysis, as shown in the Supplementary image, indicated stable results. Systematically removing studies one at a time fails to make heterogeneity reduce significantly. After subgroup analysis, regression analysis was conducted, as detailed in Figure 7.

#### 3.5.4 E_2_

**Figure 8:**
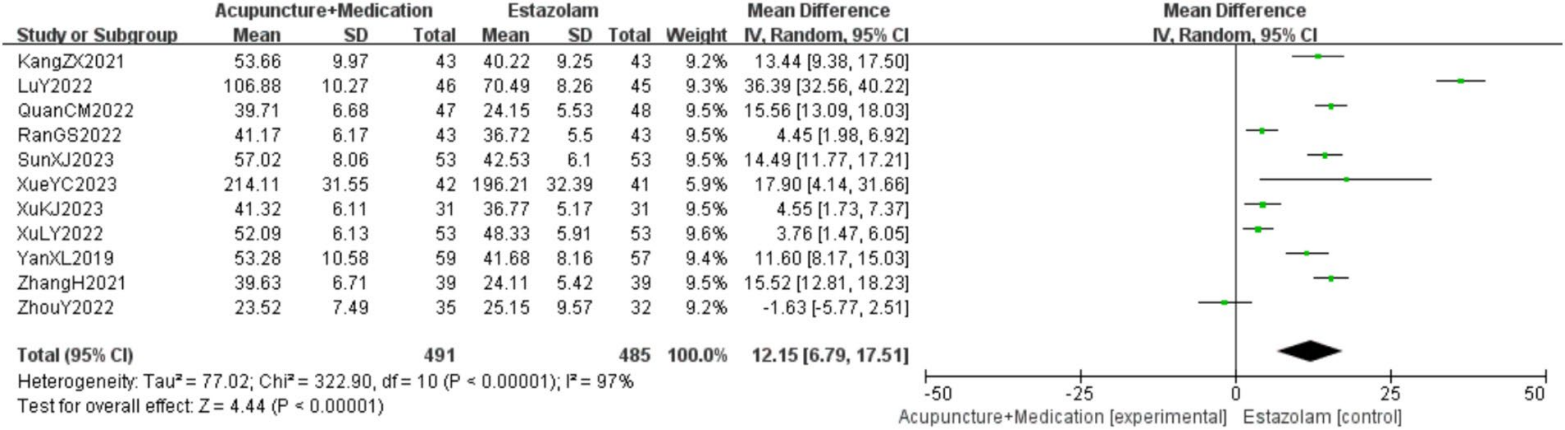
E_2_ forest plot.

A total of 976 patients in 11 studies (18–21, 23–27, 29, 30) reported the E_2_ levels of acupuncture combined with medication in treating PMI. Analysis of the extracted data revealed high heterogeneity (*p* < 0.00001; *I^2^* = 97%), so a random-effects model was selectd. The results indicated that, compared to the control group, combination therapy was more effective in improving E_2_ levels with statistical significance [MD = 12.15, 95%CI (6.79, 17.51), *Z* = 4.44, *p* < 0.00001]. Sensitivity analysis, as shown in the Supplementary image, indicated stable results. Systematically removing studies one at a time fails to make heterogeneity reduce significantly. After subgroup analysis, regression analysis was conducted, as detailed in Figure 8.

#### 3.5.5 HAMA

**Figure 9:**
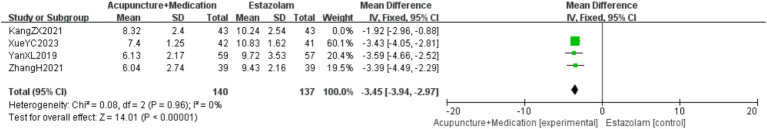
HAMA forest plot.

A total of 363 patients in 4 studies (18, 25, 26, 29) reported the HAMA scores of acupuncture combined with medication in treating PMI. Analysis of the extracted data revealed moderate heterogeneity (*p* = 0.07 < 0.1; *I^2^* = 57%), so a random-effects model was selected. The results indicated that, compared to the control group, combination therapy was more effective in improving HAMA scores with statistical significance [MD = −3.11, 95%CI (−3.82, −2.40), *Z* = 8.59, *p* < 0.00001]. Subgroup analysis did not significantly reduce heterogeneity. Sensitivity analysis, as shown in the Supplementary image, indicated stable results. Systematically removing studies one at a time (Table 4), it was found that excluding Kang (18) significantly reduced heterogeneity (*p* = 0.96 > 0.1; *I^2^* = 0%), suggesting that this study might be a source of heterogeneity. Change to a fixed-effect model [MD = −3.45, 95%CI (−3.94, −2.97), *Z* = 14.01, *p* < 0.00001], as detailed in Figure 9.

**Table 4:** Sensitivity analysis report of HAMA.

Exclusion	MD [95%CI]	*p*	*I^2^*
Kang (18)	−3.45 [−3.94, −2.97]	0.96	0%
Xue et al. (29)	−2.96 [−4.00, −1.91]	0.06	65%
Yan et al. (25)	−2.96 [−3.89, −2.03]	0.04	68%
Zhang and Zhou (26)	−3.02 [−3.98, −2.06]	0.03	70%

#### 3.5.6 KMI

**Figure 10:**
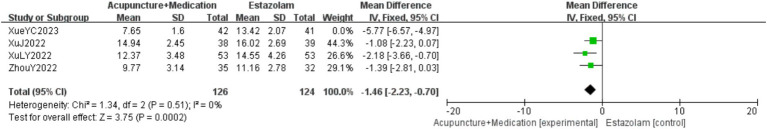
KMI forest plot.

A total of 333 patients in 4 studies (22, 23, 29, 30) reported the KMI of acupuncture combined with medication in treating PMI. Analysis of the extracted data revealed high heterogeneity (*p* < 0.00001; *I^2^* = 95%), so a random-effects model was selected. The results indicated that, compared to the control group, combination therapy was more effective in improving KMI with marginal statistical significance [MD = −2.64, 95%CI (−5.24, −0.03), *Z* = 1.98, *p =* 0.05]. Subgroup analysis did not significantly reduce heterogeneity. Sensitivity analysis, as shown in the Supplementary image, indicated stable results. Systematically removing studies one at a time (Table 5), it was found that excluding Xue et al. (29) significantly reduced heterogeneity (*p* = 0.51 > 0.1; *I^2^* = 0%), suggesting that this study might be a source of heterogeneity. Change to a fixed-effect model [MD = −1.46, 95%CI (−2.23, −0.70), *Z* = 3.75, *p =* 0.0002 < 0.05], as detailed in Figure 10.

**Table 5:** Sensitivity analysis report of KMI.

Exclusion	MD [95%CI]	*p*	*I^2^*
Xue et al. (29)	−1.46 [−2.23, −0.70]	0.51	0%
Xu (22)	−3.16 [−6.18, −0.15]	<0.00001	95%
Xu and Zhao (23)	−2.78 [−6.13, 0.58]	<0.00001	96%
Zhou (30)	−3.04 [−6.24, 0.16]	<0.00001	96%

#### 3.5.7 TCMS

**Figure 11:**
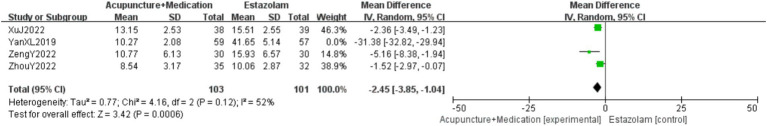
TCMS forest plot.

A total of 320 patients in 4 studies (22, 25, 28, 30) reported the TCMS scores of acupuncture combined with medication in treating PMI. Analysis of the extracted data revealed high heterogeneity (*p* < 0.00001; *I^2^* = 100%), so a random-effects model was selected. The results indicated that, compared to the control group, combination therapy was more effective in improving TCM scores but not statistically significant [MD = −10.11, 95%CI (−25.40, 5.17), *Z* = 1.30, *p* = 0.19 > 0.05]. Subgroup analysis did not significantly reduce heterogeneity. Sensitivity analysis, as shown in the Supplementary image, indicated stable results. Systematically removing studies one at a time (Table 6), it was found that excluding Yan et al. (25) reduced heterogeneity to a moderate level (*p* = 0.12 > 0.1; *I^2^* = 52%), suggesting that this study might be a source of heterogeneity. The overall effect reached statistical significance [MD = −2.45, 95%CI (−3.85, −1.04), *Z* = 3.42, *p* = 0.0006 < 0.05], as detailed in Figure 11.

**Table 6:** Sensitivity analysis report of TCMS.

Exclusion	MD [95%CI]	*p*	*I^2^*
Yan et al. (25)	−2.45 [−3.85, −1.04]	0.12	52%
Xu (22)	−12.70 [−34.44, 9.04]	<0.00001	100%
Zeng (28)	−11.75 [−30.26, 6.76]	<0.00001	100%
Zhou (30)	−12.98 [−34.19, 8.23]	<0.00001	100%

### 3.6 Subgroup analysis, sensitivity analysis and regression analysis

Two subgroups were created based on the different interventions: one for acupuncture combined with TCM and the other for acupuncture combined with WM. For outcome measures with significant heterogeneity remaining after subgroup analysis, sensitivity analysis was conducted to assess robustness, and studies were excluded one by one to identify the source of heterogeneity. Regression analysis was performed for those outcome measures where heterogeneity remained unresolved. The detailed results can be found in the Supplementary materials.

### 3.7 Assessment of publication bias

**Figure 12:**
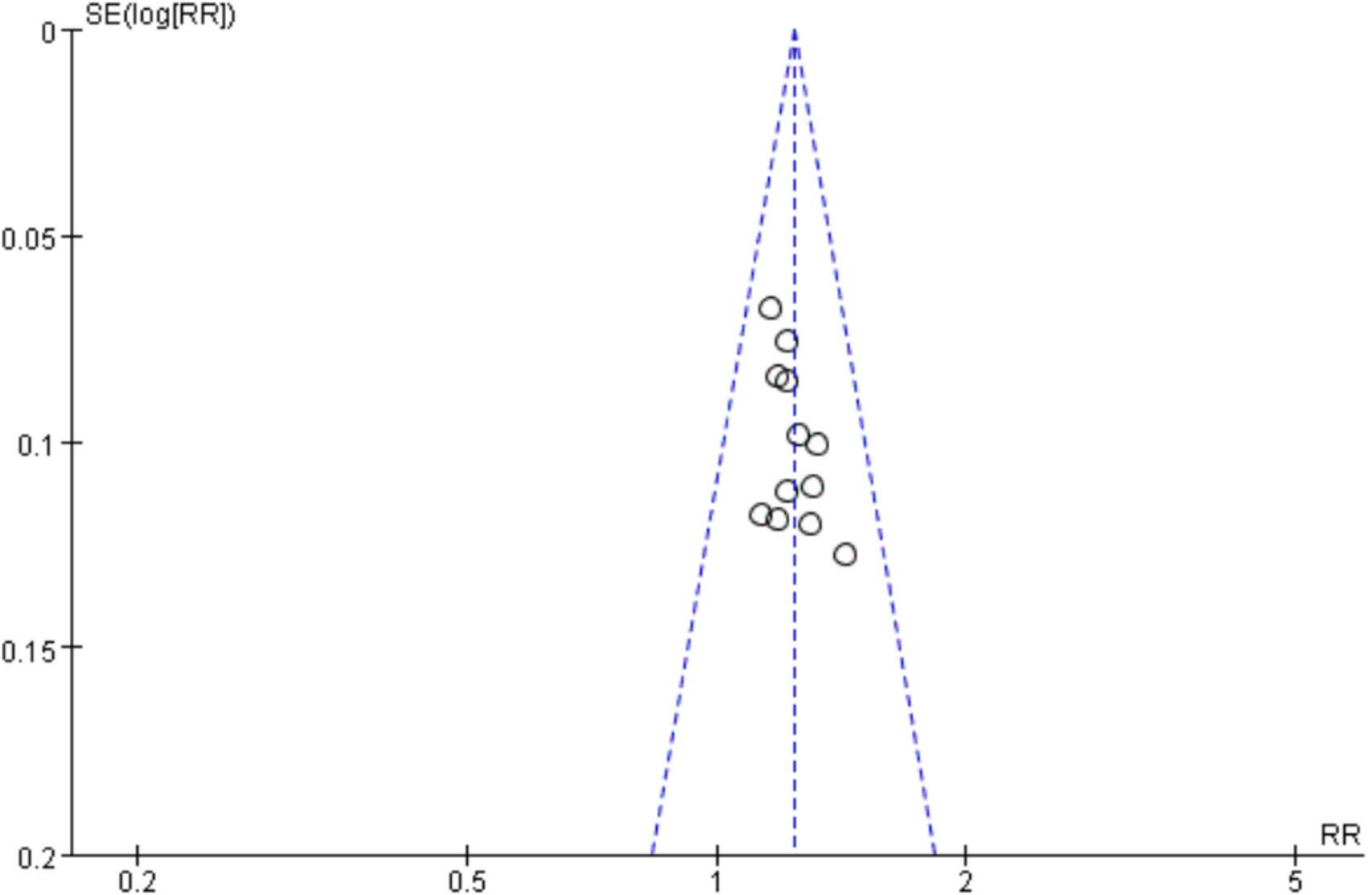
Clinical effective rate funnel plot.

**Figure 13:**
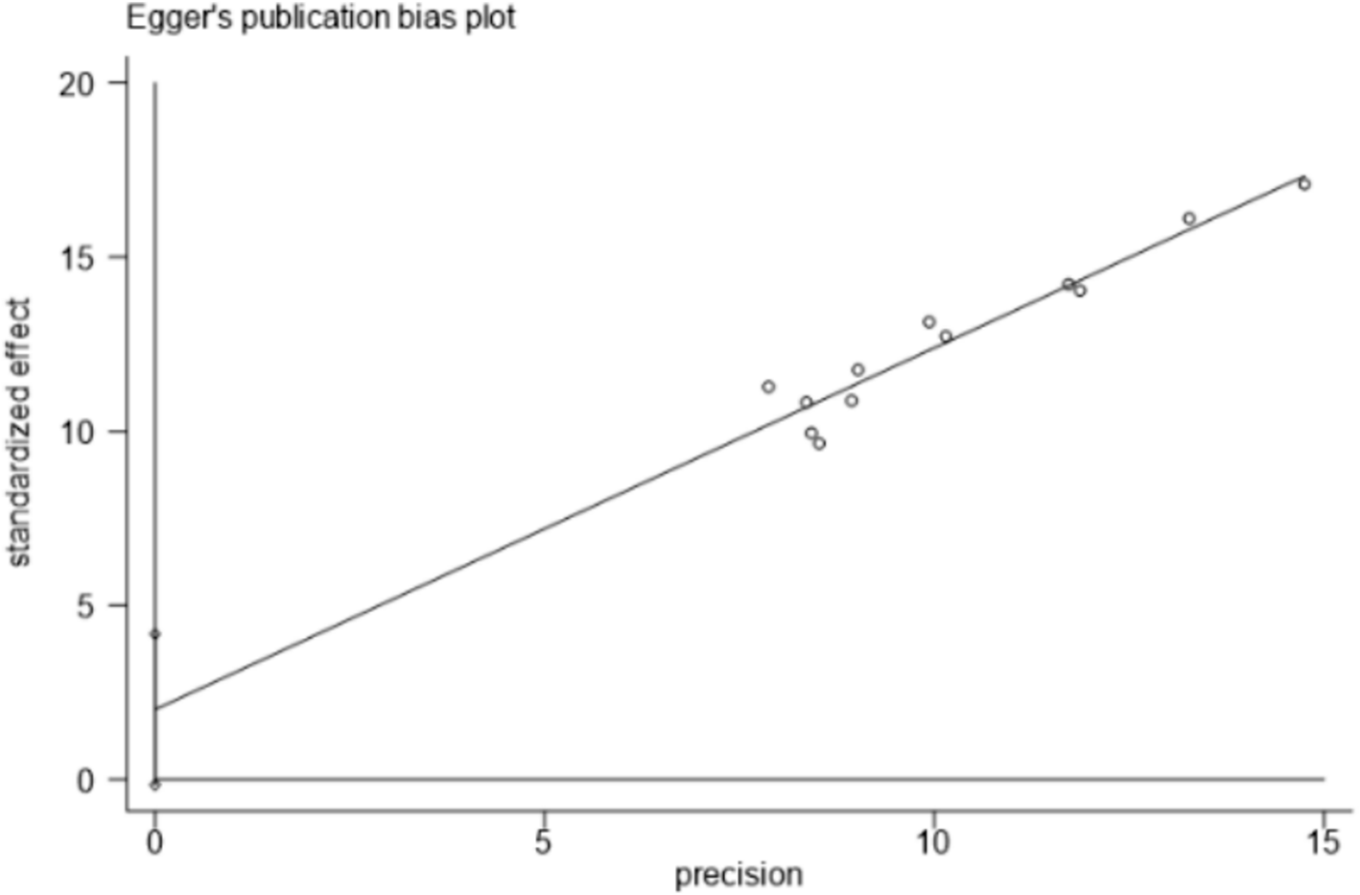
Egger test.

**Figure 14:**
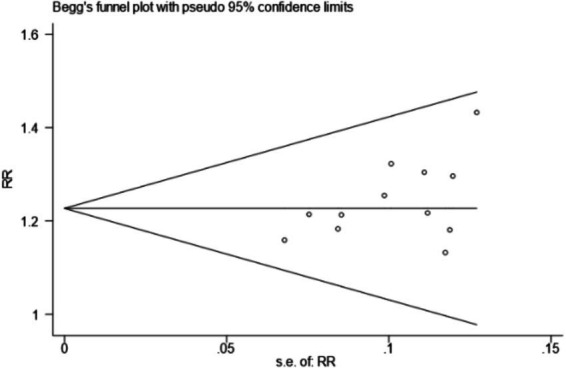
Begg test.

A funnel plot was generated using the clinical effective rate as an indicator to assess publication bias. The results showed that the scatter plot distribution was not completely symmetrical, suggesting a potential for publication bias. The Egger test using Stata 15.0 software showed that the scatter points deviated significantly from the regression line in the high precision area, indicating potential publication bias. However, further statistical testing of the regression analysis *p*-value suggested that there might not be significant publication bias (*t* = 2.07, *p* = 0.065 > 0.05). The Begg test revealed that the points were relatively evenly distributed and most points were close to the regression line, confirming that the study results were not significantly affected by publication bias, as detailed in Figures 12–14.

### 3.8 Quality of evidence grading

We utilized GRADEprofiler software to evaluate it. The assessment results showed that the quality of evidence is relatively high. Further details are provided in the Supplementary materials.

## 4 Discussion

PMI is closely related to a variety of physiological and psychological factors (2, 32). During perimenopause, ovarian function gradually decreases, with estrogen and progesterone levels fluctuate significantly, which not only affects women’s physiological functions, but also leads to a series of psychiatric symptoms such as insomnia and depression (33, 34), or even affects daily life. Sleep regulation is controlled by both lower and higher centers, and when hormonal fluctuations affect cortical function, the higher center’s regulation of the sleep center is weakened, leading to sleep structure disruption and insomnia (35, 36). In addition, the decline in estrogen may inhibit the synthesis and secretion of neurotransmitters such as 5-hydroxytryptamine (5-HT) and γ-aminobutyric acid (GABA), thereby affecting mood regulation and sleep quality (36). According to the *Inner Canon of the Yellow Emperor* (*Huangdi Neijing*), perimenopause is described as the cessation of menstruation, aligning with the concept of perimenopause. PMI falls under the category of gynecological disorders related to insomnia and is closely linked to various internal and external factors (6). The main pathogenesis, including liver and kidney deficiency, imbalance of yin and yang, deficiency of both the heart and spleen, and disharmony of qi and blood, may be closely related to changes in endocrine levels, leading to imbalances in qi and blood, emotional instability, and sleep disturbances (37). TCM adopts a syndrome differentiation approach, using acupuncture and herbal medication to regulate organ functions and improve sleep, which complements the management of hormone imbalances in WM. Recent studies have compared the distribution of Governor Meridian vessels with the hypothalamic–pituitary–adrenal (HPA) axis, finding that electroacupuncture stimulation at points along the Governor Meridian can effectively reduce HPA related hormones [cortisol (CORT), adrenocorticotrophin (ACTH), and corticotropin-releasing hormone (CRH)] and hypothalamic–pituitary-ovarian (HPO) axis related hormones (FSH and LH), alleviating clinical symptoms of PMI (38). In terms of herbal medication, commonly used formulas include Liuwei Dihuang Pill and Jiawei Yigan Powder, focusing on nourishing kidney yin, replenishing liver blood, and calming the mind, all showing great efficacy for PMI (39). Li (15) performed a meta-analysis, demonstrating that acupuncture combined with TCM is more effective and safe in treating PMI. In contrast, WM commonly uses sedative-hypnotic drugs, but long-term use can lead to tolerance and cognitive impairments such as memory decline (40). One study found that nighttime pressure on the Shenmen point (HT_7_) on the wrist increased the rhythm of urinary melatonin metabolites within 24 h without adverse effects, suggesting that acupoint stimulation might naturally improve sleep quality in insomnia patients and could be more advantageous than using WM alone (41). Zhao (42) confirmed through a meta-analysis that acupuncture combined with WM is highly effective. The mechanisms of acupuncture combined with medication for treating PMI are multifaceted, with fewer side effects, making this method simpler and potentially suitable as an alternative therapy for widespread clinical use (43).

For the definition and specific measurement methods of the outcome measure labeled “Clinical Effective Rate,” 7 studies (18, 20, 23, 25, 28–30) employed the “Guidelines for Clinical Research of New Traditional Chinese Medicines,” 1 study (19) utilized the “Guidelines for Clinical Research of New Traditional Chinese Medicine for Treating Insomnia,” 1 study (21) used the “Chinese Guidelines for the Diagnosis and Treatment of Insomnia,” and 1 study (22) incorporated both sets of guidelines. However, 2 studies (24, 26) did not specify the use of any internationally recognized evaluation criteria, which may introduce additional heterogeneity and lead to inconsistencies in treatment outcome assessments. To improve the comparability of future studies and the robustness of meta-analysis results, it is essential to establish clear, standardized, and internationally recognized criteria for evaluating efficacy.

Heterogeneity management revealed that the group variable significantly affected FSH and E_2_ levels, while the subgroups showed no significant difference in their impact on PSQI and LH levels, suggesting other potential sources of heterogeneity. The treatment approaches of acupuncture combined with TCM and acupuncture combined with WM involve significant differences in mechanisms and implementation, which may contribute to heterogeneous treatment effects. In addition, patient physiological characteristics and external factors (geographical and cultural differences, treatment adherence, and genetic background) may also impact the stability of the results. To more accurately identify and quantify the sources of heterogeneity, subsequent research may consider finer subgroup divisions and standardized assessment tools, or, when appropriate, the use of mixed-effects models and Bayesian methods for further exploration. By taking these factors into account, a more comprehensive understanding of the variability in intervention effects can be achieved, providing a basis for the individualized development of treatment plans.

This study has the following advantages: (1) This article comprehensively assessed the effectiveness and safety of combination therapy in treating PMI; (2) The search scope covered 8 databases, including both Chinese and English, making the results relatively comprehensive and extensive; (3) This study used established clinical outcome indicators.

This study still has some limitations: (1) The studies included are all in Chinese, which may introduce language bias and limit the generalizability of the findings to non-Chinese populations, especially given the ongoing shortage of high-quality blinded clinical trials; (2) During the data collection process, the same indicators in different studies use different units, and there are no internationally recognized conversion standards between some of these units, which poses difficulties for analysis; (3) Some indicators still show significant heterogeneity, and other potential subgroups should be considered; (4) Due to the lack of sufficient data and supporting literature, a more in-depth discussion on the relationship between effect size and the Minimal Clinically Important Difference (MCID) was not conducted; (5) Insufficient attention has been given to the high homogeneity of the included studies.

Considering the above limitations, future research should select databases in multiple languages to reduce bias caused by language differences; adopt strict inclusion criteria and refine subgroups; consider large-scale, multicenter RCTs to assess the effectiveness of integrated treatments in a more comprehensive manner; and expand data sources to enable a deeper exploration of the relationship between effect size and the Minimal Clinically Important Difference (MCID). By implementing these strategies, the clinical value of acupuncture combined with medication can be further established, promoting the development of individualized treatment plans and ultimately enhancing overall patient outcomes and quality of life.

## Data Availability

The original contributions presented in the study are included in the article/Supplementary material, further inquiries can be directed to the corresponding author.

## References

[1] Harlow SD Gass M Hall JE Lobo R Maki P Rebar RW . STRAW+ 10 collaborative group. Executive summary of the stages of reproductive aging workshop+ 10: addressing the unfinished agenda of staging reproductive aging. J Clin Endocrinol Metab. (2012) 97:1159–68. doi: 10.1210/jc.2011-3362, PMID: 22344196 PMC3319184

[2] Dennerstein L Dudley EC Hopper JL Guthrie JR Burger HG. A prospective population-based study of menopausal symptoms. Obstet Gynecol. (2000) 96:351–8. doi: 10.1016/s0029-7844(00)00930-3 10960625

[3] Santoro N Epperson CN Mathews SB. Menopausal symptoms and their management. Endocrinol Metab Clin. (2015) 44:497–515. doi: 10.1016/j.ecl.2015.05.001, PMID: 26316239 PMC4890704

[4] Ruan X Cui Y Du J Jin F Mueck AO. Prevalence of climacteric symptoms comparing perimenopausal and postmenopausal Chinese women. J Psychosom Obstet Gynecol. (2017) 38:161–9. doi: 10.1080/0167482X.2016.1244181, PMID: 27766930

[5] Rasgon N Shelton S Halbreich U. Perimenopausal mental disorders: epidemiology and phenomenology. CNS Spectr. (2005) 10:471–8. doi: 10.1017/S1092852900023166, PMID: 15908901

[6] Sun D Shao H Li C Tao M. An analysis of the main reasons that perimenopausal and postmenopausal women in China have for seeking outpatient treatment and factors influencing their symptoms: a single-center survey. Clin Exp Obstet Gynecol. (2015) 42:146–51. doi: 10.12891/ceog1744.2015, PMID: 26054107

[7] Carpenter J Gass ML Maki PM Newton KM Pinkerton JV Taylor M . Nonhormonal management of menopause-associated vasomotor symptoms: 2015 position statement of the North American Menopause Society. Menopause. (2015) 22:1155–74. doi: 10.1097/GME.0000000000000546 26382310

[8] Choi E Lee JK Baek JK Chung Y Kim H Yun BH . Hormone replacement therapy and breast cancer incidence in Korean women. Maturitas. (2024) 183:107946. doi: 10.1016/j.maturitas.2024.107946, PMID: 38412593

[9] Simon T Jaillon P. Hormone replacement therapy and cardiovascular risk in menopausal women. Arch Mal Coeur Vaiss. (2001) 94:132–8. PMID: 11265551

[10] Canonico M Oger E Plu-Bureau G Conard J Meyer G Lévesque H . Hormone therapy and venous thromboembolism among postmenopausal women: impact of the route of estrogen administration and progestogens: the ESTHER study. Circulation. (2007) 115:840–5. doi: 10.1161/CIRCULATIONAHA.106.642280, PMID: 17309934

[11] Ovsyannikova TV Makarov IO Kulikov IA. Clinical efficacy of non-hormonal methods of therapy in perimenopausal women. Obstet Gynecol Reprod. (2016) 7:26–9.

[12] Shi R Meng W Liu Z Xue W Chen X Deng Y. Exploring acupuncture as a treatment for insomnia in perimenopausal women with stable angina pectoris: a protocol for a randomized, double-blind, placebo-controlled clinical trial. PLoS One. (2024) 19:e0301827. doi: 10.1371/journal.pone.0301827, PMID: 38635812 PMC11025937

[13] Gingrich PM Fogel CI. Herbal therapy use by perimenopausal women. J Obstet Gynecol Neonatal Nurs. (2003) 32:181–9. doi: 10.1177/0884217503251706, PMID: 12685669

[14] Cui CH Qu Z Chen YJ Fu SG. Clinical study on acupuncture combined with medicine for treatment of insomnia in Perimenopausal women with kidney deficiency and liver stagnation type. Chin Arch Tradit Chin Med. (2023) 41:78–81. doi: 10.13193/j.issn.1673-7717.2023.11.018

[15] Li Z Yin S Feng J Gao X Yang Q Zhu F. Acupuncture combined with Chinese herbal medicine in the treatment of perimenopausal insomnia: a systematic review and meta-analysis. Medicine. (2023) 102:e35942. doi: 10.1097/MD.0000000000035942, PMID: 37960761 PMC10637479

[16] Page MJ JE MK Bossuyt PM Boutron I Hoffmann TC Mulrow CD . The PRISMA 2020 statement: an updated guideline for reporting systematic reviews. BMJ. (2021) 372:n71. doi: 10.1136/bmj.n71, PMID: 33782057 PMC8005924

[17] Higgins JP Altman DG Gøtzsche PC Jüni P Moher D Oxman AD . The Cochrane Collaboration’s tool for assessing risk of bias in randomised trials. BMJ. (2011) 343:d5928. doi: 10.1136/bmj.d5928, PMID: 22008217 PMC3196245

[18] Kang ZX. Clinical study on acupuncture combined with modified Xiangfu decoction for treating Perimenopausal insomnia with liver qi stagnation syndrome. Pract Clin J Integ Tradition Chin Western Med. (2021) 21:35–6. doi: 10.13638/j.issn.1671-4040.2021.01.014

[19] Lu Y Li CC Gao T Guo YY. Clinical observation on treatment of Peri-menopausal insomnia with Baizi Yangxin decoction and acupuncture. J Pract Tradition Chin Int Med. (2022) 36:8–10. doi: 10.13729/j.issn.1671-7813.Z20210186

[20] Quan CM Yan QL. The effect of the Ganmai Dazao decoction plus Yuanluo acupuncture on female menopausal insomnia and its influence on serum neurotransmitter levels. Clin J Chin Med. (2022) 14:63–6. doi: 10.3969/j.issn.1674-7860.2022.25.017

[21] Sun XJ Zhao KN Liu BX Chen L. Clinical observation of acupuncture combined with heat clearing and mind calming decoction in the treatment of perimenopausal insomnia. Guangxi J Tradition Chin Med. (2023) 46:27–9.

[22] Xu J. Clinical study of Wendan decoction combined with head acupuncture and Limbal acupuncture in the treatment of syndrome of phlegm-heat based Perimenopausal insomnia. Nanchang, China: Jiangxi University of Chinese Medicine (2022).

[23] Xu LY Zhao YR. Therapeutic observation of Wen'an Shenyangxue decoction combined with Jinsan needle in the treatment of perimenopausal insomnia patients. Clin Educ Gen Pract. (2022) 20:425–8. doi: 10.13558/j.cnki.issn1672-3686.2022.005.012

[24] Xu KJ. The clinical effect of Suanzaoren decoction combined with acupuncture on perimenopausal insomnia. Mat Child Nurs. (2023) 3:5034–6. doi: 10.3969/j.issn.2097-0838.2023.20.086

[25] Yan XL Yu YD Yang DD. Clinical efficacy of acupuncture combined with modified Xiangfu decoction in treatment of menopausal insomnia cause by liver qi stagnation. China J Chin Mat Med. (2020) 45:1460–4. doi: 10.19540/j.cnki.cjcmm.20191010.501, PMID: 32281361

[26] Zhang H Zhou Q. Efficacy of Baihe Dihuang decoction combined with Zhenjing Anshen acupuncture in the treatment of female menopausal insomnia and influence on anxiety and depression. World J Integ Tradition Western Med. (2021) 16:405–9. doi: 10.13935/j.cnki.sjzx.210303

[27] Ran GS Wang Y. Clinical study on supplemented sour jujube decoction combined with acupuncture and Moxibustion in treating Perimenopausal insomnia. Henan Tradition Chin Med. (2022) 42:1644–7. doi: 10.16367/j.issn.1003-5028.2022.11.0348

[28] Zeng Y. Clinical observation of Perimenopausal sub-health insomnia by scalp acupuncture combined with Tiaojing Anshen decoction. Luzhou, China: Southwest Medical University (2022).

[29] Xue YC Chen L Hua CF. Clinical effect of Jieyu Jianpi acupuncture combined with estazolam tablets on perimenopausal insomnia of liver spleen deficiency. Hebei J Tradit Chin Med. (2023) 45:1712–1716+1720. doi: 10.3969/j.issn.1002-2619.2023.10.030

[30] Zhou Y. Clinical observation of superficial needling on auricular point combine with Estazolam in the treatment of Perimenopausal insomnia with heart-kidney disharmony. Fuzhou, China: Fujian University of Traditional Chinese Medicine (2022).

[31] Zhu SP Li PP Zhu XL. Observation of the therapeutic effect of Tiao Du an Shen acupuncture method on Perimenopausal insomnia. Modern J Integ Tradition Chin Western Med. (2016) 25:2885–8. doi: 10.3969/j.issn.1008-8849.2016.26.011

[32] Smith RL Flaws JA Mahoney MM. Factors associated with poor sleep during menopause: results from the midlife Women's health study. Sleep Med. (2018) 45:98–105. doi: 10.1016/j.sleep.2018.01.012, PMID: 29680438 PMC5918428

[33] Haufe A Baker FC Leeners B. The role of ovarian hormones in the pathophysiology of perimenopausal sleep disturbances: a systematic review. Sleep Med Rev. (2022) 66:101710. doi: 10.1016/j.smrv.2022.101710, PMID: 36356400

[34] de Zambotti M Colrain IM Baker FC. Interaction between reproductive hormones and physiological sleep in women. J Clin Endocrinol Metab. (2015) 100:1426–33. doi: 10.1210/jc.2014-3892, PMID: 25642589 PMC4399298

[35] Freedman RR Roehrs TA. Sleep disturbance in menopause. Menopause. (2007) 14:826–9. doi: 10.1097/gme.0b013e3180321a22, PMID: 17486023

[36] Moline ML Broch L Zak R Gross V. Sleep in women across the life cycle from adulthood through menopause. Sleep Med Rev. (2003) 7:155–77. doi: 10.1053/smrv.2001.0228, PMID: 12628216

[37] Zhao FY Fu QQ Spencer SJ Kennedy GA Conduit R Zhang WJ . Acupuncture: a promising approach for comorbid depression and insomnia in perimenopause. Nat Sci Sleep. (2021) 13:1823–63. doi: 10.2147/NSS.S332474, PMID: 34675729 PMC8520448

[38] Tang Y Zheng QC Huang JF Chen Y. Effect of acupuncture on the endocrine axis in patients with perimenopausal insomnia: a case series study. World J Acupunct Moxibustion. (2023) 33:97–101. doi: 10.1016/j.wjam.2022.05.007

[39] Yao ZY Chen H. Recent development of Peri-menopausal insomnia treated by traditional Chinese medicine. Chin Arch Tradit Chin Med. (2014) 32:627–9. doi: 10.13193/j.issn.1673-7717.2014.03.062

[40] Proserpio P Marra S Campana C Agostoni EC Palagini L Nobili L . Insomnia and menopause: a narrative review on mechanisms and treatments. Climacteric. (2020) 23:539–49. doi: 10.1080/13697137.2020.1799973, PMID: 32880197

[41] Nordio M Romanelli F. Efficacy of wrists overnight compression (HT 7 point) on insomniacs: possible role of melatonin? Minerva Med. (2008) 99:539–47. PMID: 19034253

[42] Zhao FY Fu QQ Kennedy GA Conduit R Wu WZ Zhang WJ . Comparative utility of acupuncture and western medication in the management of perimenopausal insomnia: a systematic review and meta-analysis. Evid Based Complement Alternat Med. (2021) 2021:5566742. doi: 10.1155/2021/5566742 33986818 PMC8093060

[43] Su HW Chen HT Kao CL Hung KC Lin YT Liu PH . Efficacy and safety of herbal medicine combined with acupuncture in pediatric epilepsy treatment: a meta-analysis of randomized controlled trials. PLoS One. (2024) 19:e0303201. doi: 10.1371/journal.pone.0303201, PMID: 38723054 PMC11081325

