# Supplementary material for: The effectiveness and safety of acupuncture combined with medication in the treatment of perimenopausal insomnia: a systematic review and meta-analysis
Source: Front Neurol. 2025 Mar 13;16:1476719. doi: 10.3389/fneur.2025.1476719 (PMC11966447; doi:10.3389/fneur.2025.1476719)
Supplement: Supplementary file 1 [file Data_Sheet_1.ZIP › Basic characteristics of the studies.docx]

| **Study** | **Experimental treatment** | **Control**  **treatment** | **Sample**  **size (E/C)** | **Age[mean**  **(SD)] (E/C)** | **Course[mean**  **(SD)] (E/C)** | **Acupuncture points** | **Medication dosages (per dose)** |
| --- | --- | --- | --- | --- | --- | --- | --- |
| KangZX2021^[25]^ | Acupuncture and Xiang Fu Tang | Estazolam | 43/43 | 50.45(3.92)y/49.30(3.15)y | 2.91(0.56)y/3.11(0.73)y | BL_62_, KI_6_, SP_6_, BL_20_, ST_36_ | 1-2mg |
| LuY2022^[26]^ | Acupuncture and Bai Zi Yang Xin Tang | Estazolam | 46/45 | 46.13(-)y/46.13(-)y | 5.1(0.7)y/4.6(0.8)y | DU_20_, EX-HN_1_, BL_15_, BL_23_, ST_25_, RN_4_, EX-CA_1_, SP_6_ | 2mg |
| QuanCM2022^[27]^ | Acupuncture and Gan Mai Da Zao Tang | Estazolam | 47/48 | 49.76(2.71)y49.14(2.93)y | 2.25(0.78)y/2.12(0.67)y | DU_20_, EX-HN_1_, HT_5_, HT_7_, SP_6_, KI_3_, KI_4_, BL_62_, KI_6_ | 1-2mg |
| SunXJ2023^[28]^ | Acupuncture and Qing Re An Shen Tang | Estazolam | 53/53 | 51.62(3.11)y/51.66(3.12)y | 27.82(1.21)m/27.84(1.23)m | DU_20_, EX-HN_3_, GB_20_, KI_6_, HT_7_, SP_6_, BL_15_, AnMian, PC_6_ | 2mg |
| XuJ2022^[29]^ | Acupuncture and Wen Dan Tang | Estazolam | 38/39 | 50.81(3.04)y/50.18(3.05)y | 11.13(4.85)m/11.52(4.74)m | DU_20_, EX-HN_1_, DU_24_, AnMian, HT_7_, PC_6_, ST_40_, SP_6_ | 1mg |
| XuLY2022^[30]^ | Acupuncture and Wen An Shen Yang Xue Tang | Estazolam | 53/53 | 51.01(5.22)y/51.24(5.33)y | 11.89(2.11)m/12.05(2.16)m | 1.5 cun anterior, posterior, left, and right to DU_20_, 0.5 cun above EX-HN_3_, 0.5 cun above GB_14_ on the left and right, DU_24_, Bilateral GB_13_ | 1mg |
| XuKJ2023^[31]^ | Acupuncture and Suan Zao Ren Tang | Estazolam | 31/31 | 51.82(3.94)y/50.88(4.88)y | 8.24(3.76)m/7.58(3.42)m | HT_7_, RN_4_, AnMian, DU_20_, PC_6_, EX-HN_5_, RN_12_ | - |
| YanXL2019^[32]^ | Acupuncture and Xiang Fu Tang | Estazolam | 59/57 | 50.8(7.6)y/49.6(7.2)y | 3.1(0.5)y/2.9(0.4)y | EX-HN_1_, AnMian, HT_7_, SP_6_, BL_18_, BL_13_, GB_20_, ST_36_ | 1mg |
| ZhangH2021^[33]^ | Acupuncture and Bai He Di Huang Tang | Estazolam | 39/39 | 52.76(2.81)y/52.14(2.63)y | 2.75(0.77)y/2.09(0.64)y | DU_20_, HT_7_, DU_24_, EX-HN_1_, GB_13_, PC_6_, SP_6_ | 1-2mg |
| RanGS2022^[34]^ | Acupuncture and Suan Zao Ren Tang | Estazolam | 43/43 | 50.63(7.59)y/50.51(7.57)y | 10.72(1.60)m/10.52(1.57)m | DU_20_, HT_7_, AnMian, EX-HN_5_, RN_4_, PC_6_, RN_12_, RN_10_ | - |
| ZengY2022^[35]^ | Acupuncture and Tiao Jing An Shen Tang | Estazolam | 30/30 | 50.70(3.12)y/50.97(3.17)y | 11.02(3.19)m/10.63(2.92)m | the lower 2/5 of the vasomotor area, the upper 1/5 of the sensory area, GB_4_-GB_6_, GB_9_-SJ_20_, DU_24_-DU_20_ | 1mg |
| XueYC2023^[36]^ | Acupuncture and Estazolam | Estazolam | 42/41 | 48.35(2.37)y/47.75(3.10)y | 7.34(1.63)m/8.02(1.46)m | EX-HN_1_, AnMian, DU_20_, BL_62_, LI_4_, ST_40_, LR_14_, LR_2_, LR_3_, BL_18_, KI_6_, SP_6_, ST_36_ | 2mg |
| ZhouY2022^[37]^ | Acupuncture and Estazolam | Estazolam | 35/32 | 50.37(2.47)y/49.71(2.71)y | 7.89(2.91)m/7.94(2.96)m | HT_7_, Bilateral auricular points: Heart, Kidney, Sympathetic, Endocrine, Subcortex | 1mg |
| ZhuSP2016^[38]^ | Acupuncture and Estazolam | Estazolam | 37/37 | 49.86(3.15)y/49.27(3.58)y | 2.99(4.24)y/2.97(3.42)y | DU_20_, DU_24_, EX-HN_1_, AnMian, HT_7_, LR_3_, KI_3_, RN_12_, ST_25_, SP_9_ | 1mg |

Note: E=Experimental Group; C=Control Group; SD=Standard Deviation; P=Perimenopause; I=Insomnia; w=week(s); m=month(s); y=year(s); AEIR= Adverse Events Incidence Rate; PSQI=Pittsburgh Sleep Quality Index; LH=Luteinizing Hormone; FSH=Follicle-Stimulating Hormone; E_2_=Estradiol; HAMA=Hamilton Anxiety Scale; KMI=Kupperman Menopausal Index; TCMS=Traditional Chinese Medicine Syndromes

| **Study** | **Diagnostic criteria (P/I)** | **Duration** | **Outcome measures** |
| --- | --- | --- | --- |
| KangZX2021^[25]^ | ⑧ | 4w | Efficiency, PSQI, LH, FSH, E_2_, HAMA |
| LuY2022^[26]^ | ①⑦ | 4w | Efficiency, PSQI, FSH, E_2_ |
| QuanCM2022^[27]^ | ②⑫ | 1m | Efficiency, PSQI, LH, FSH, E_2_ |
| SunXJ2023^[28]^ | ⑪ | 2m | Efficiency, PSQI, LH, FSH, E_2_ |
| XuJ2022^[29]^ | ①⑥ | 4w | Efficiency, PSQI, KMI, TCMS |
| XuLY2022^[30]^ | - | 30d | Efficiency, PSQI, LH, FSH, E_2_, KMI, AEIR |
| XuKJ2023^[31]^ | - | - | Efficiency, PSQI, LH, FSH, E_2_, AEIR |
| YanXL2019^[32]^ | ⑨ | 16w | Efficiency, PSQI, LH, FSH, E_2_, HAMA, AEIR, TCMS |
| ZhangH2021^[33]^ | ②⑫ | 4w | Efficiency, PSQI, LH, FSH, E_2_, HAMA, AEIR |
| RanGS2022^[34]^ | ③⑫ | 4w | PSQI, LH, FSH, E_2_, AEIR |
| ZengY2022^[35]^ | ⑦⑬ | 3w | Efficiency, PSQI, TCMS |
| XueYC2023^[36]^ | ④⑦ | 4w | Efficiency, PSQI, LH, FSH, E_2_, HAMA, KMI, AEIR |
| ZhouY2022^[37]^ | ⑤⑩ | 4w | Efficiency, PSQI, FSH, E_2_, KMI, TCMS |
| ZhuSP2016^[38]^ | ⑦ | 4w | PSQI |

Note: ①Obstetrics and Gynecology ②Obstetrics and Gynecology, 2008:14. ③Chinese Obstetrics and Gynecology, 3rd ed, 2014:2537. ④Obstetrics and Gynecology, 6th ed, 2004:9. ⑤Obstetrics and Gynecology, 2018:365. ⑥Classification and Diagnostic Criteria of Mental Disorders in China ⑦China Classification and Diagnostic Criteria of Mental Disorders, 3rd ed, 2001:118-119. ⑧Key Changes in the 5th Edition of the Diagnostic and Statistical Manual of Mental Disorders (DSM-5), 2013, 23(4):289-290. ⑨Diagnostic and statistical mannual of mental disorders, 4th ed, text version ( DSM-IV), 2000. ⑩Diagnosing the Diagnostic and Statistical Manual of Mental Disorders: Fifth Edition, 2018:193. ⑪Guidelines for the Diagnosis and Treatment of Insomnia in China, 2017, 97(24):1844-1856. ⑫Guidelines for the Diagnosis and Treatment of Insomnia in Adults in China (2017 Edition), 2018, 51(5):324-335. ⑬Clinical Diagnosis and Treatment Guidelines: Obstetrics and Gynecology Volume, 2009.
