# Supplementary material for: The effectiveness and safety of acupuncture combined with medication in the treatment of perimenopausal insomnia: a systematic review and meta-analysis
Source: Front Neurol. 2025 Mar 13;16:1476719. doi: 10.3389/fneur.2025.1476719 (PMC11966447; doi:10.3389/fneur.2025.1476719)
Supplement: Supplementary file 1 [file Data_Sheet_1.ZIP › Regression analysis/Regression analysis.docx]

| _ES | Coef. | Std. Err. | t | P>\|t\| | [95% Conf. Interval] |
| --- | --- | --- | --- | --- | --- |
| group | -1.199186 | 1.383622 | -0.87 | 0.403 | -4.21384 1.815468 |
| _cons | -.6527138 | 1.766575 | -0.37 | 0.718 | -4.501751 3.196323 |

PSQI-Regression analysis

| _ES | Coef. | Std. Err. | t | P>\|t\| | [95% Conf. Interval] |
| --- | --- | --- | --- | --- | --- |
| group | .14219 | .9920144 | 0.14 | 0.890 | -2.203551 2.487931 |
| _cons | -.7770891 | 1.145693 | -0.68 | 0.519 | -3.486223 1.932044 |

LH-Regression analysis

| _ES | Coef. | Std. Err. | t | P>\|t\| | [95% Conf. Interval] |
| --- | --- | --- | --- | --- | --- |
| group | 1.189029 | .7211004 | 1.65 | 0.134 | -.442213 2.820272 |
| _cons | -2.750244 | .8975105 | -3.06 | 0.013 | -4.780554 -.7199341 |

FSH-Regression analysis

| _ES | Coef. | Std. Err. | t | P>\|t\| | [95% Conf. Interval] |
| --- | --- | --- | --- | --- | --- |
| group | -1.54874 | .8015353 | -1.93 | 0.085 | -3.361939 .2644588 |
| _cons | 3.283903 | .9982155 | 3.29 | 0.009 | 1.025783 5.542023 |

E_2_-Regression analysis
