# Supplementary figures and images for: The effectiveness and safety of acupuncture combined with medication in the treatment of perimenopausal insomnia: a systematic review and meta-analysis
Source: Front Neurol. 2025 Mar 13;16:1476719. doi: 10.3389/fneur.2025.1476719 (PMC11966447; doi:10.3389/fneur.2025.1476719)

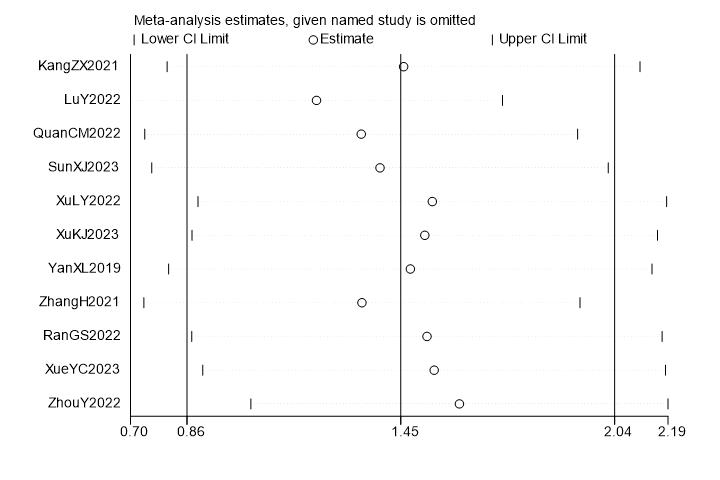

Supplement: Supplementary file 1 [file Data_Sheet_1.ZIP › Sensitivity analysis/E2-Sensitivity analysis.jpg]

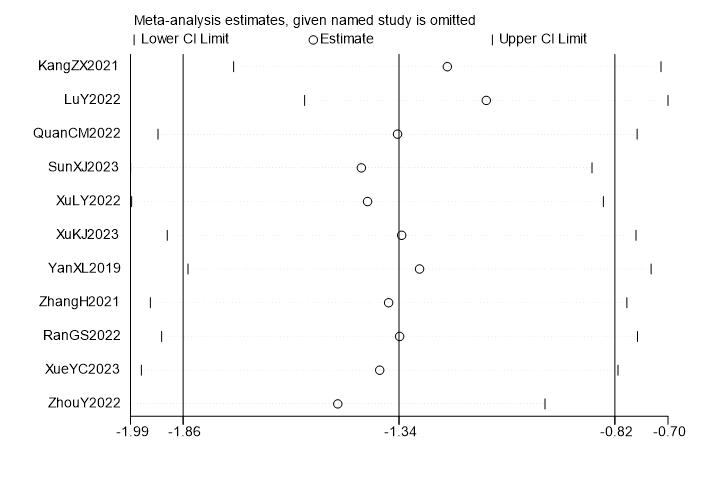

Supplement: Supplementary file 1 [file Data_Sheet_1.ZIP › Sensitivity analysis/FSH-Sensitivity analysis.jpg]

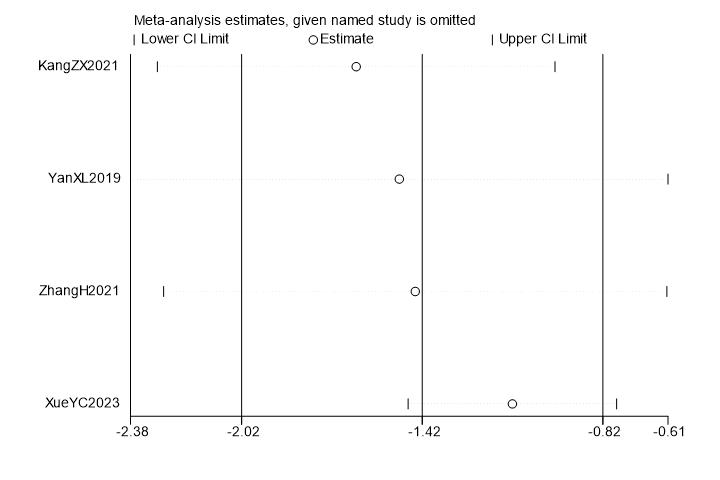

Supplement: Supplementary file 1 [file Data_Sheet_1.ZIP › Sensitivity analysis/HAMA-Sensitivity analysis.jpg]

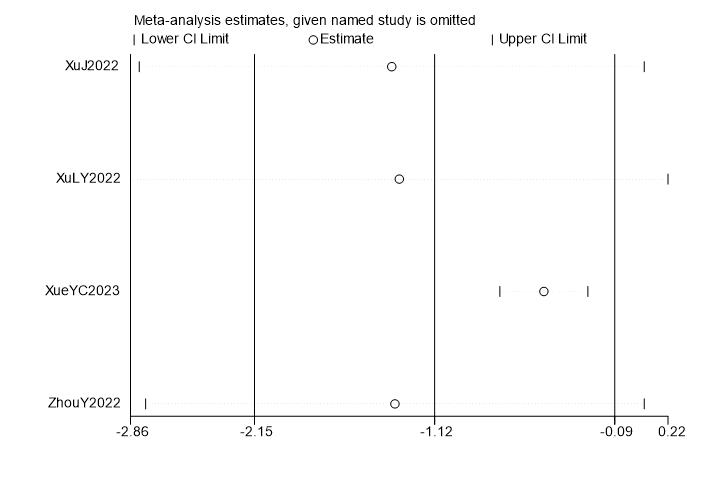

Supplement: Supplementary file 1 [file Data_Sheet_1.ZIP › Sensitivity analysis/KMI-Sensitivity analysis.jpg]

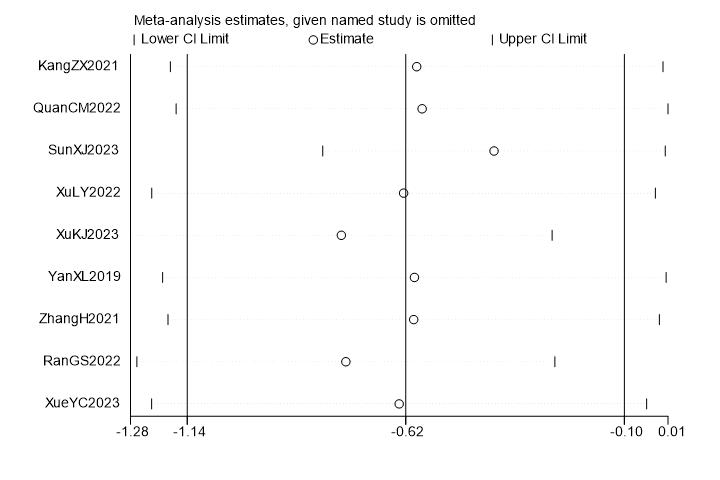

Supplement: Supplementary file 1 [file Data_Sheet_1.ZIP › Sensitivity analysis/LH-Sensitivity analysis.jpg]

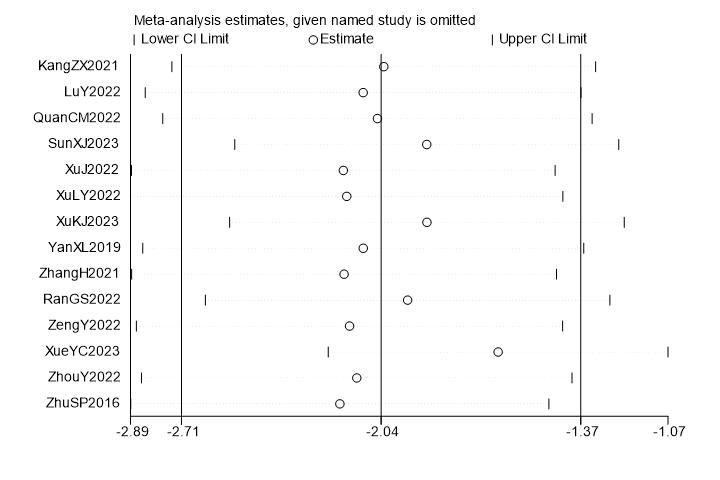

Supplement: Supplementary file 1 [file Data_Sheet_1.ZIP › Sensitivity analysis/PSQI-Sensitivity analysis.jpg]

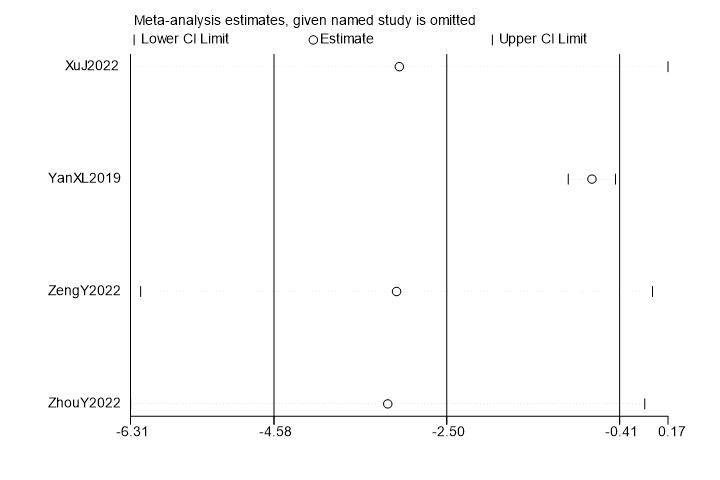

Supplement: Supplementary file 1 [file Data_Sheet_1.ZIP › Sensitivity analysis/TCMS-Sensitivity analysis.jpg]

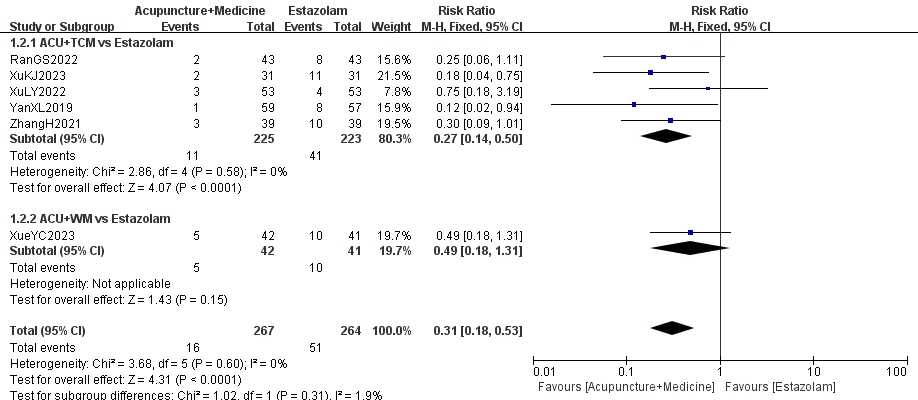

Supplement: Supplementary file 1 [file Data_Sheet_1.ZIP › Subgroup analysis/Adverse Events-Subgroup analysis.jpg]

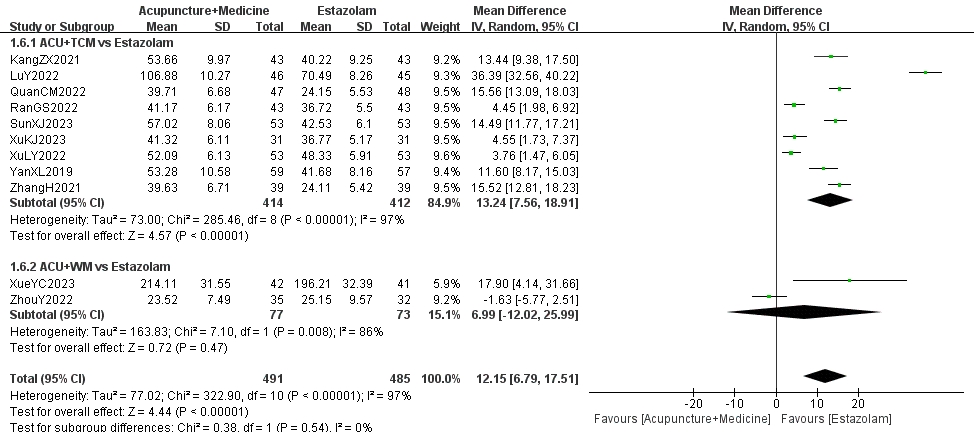

Supplement: Supplementary file 1 [file Data_Sheet_1.ZIP › Subgroup analysis/E2-Subgroup analysis.jpg]

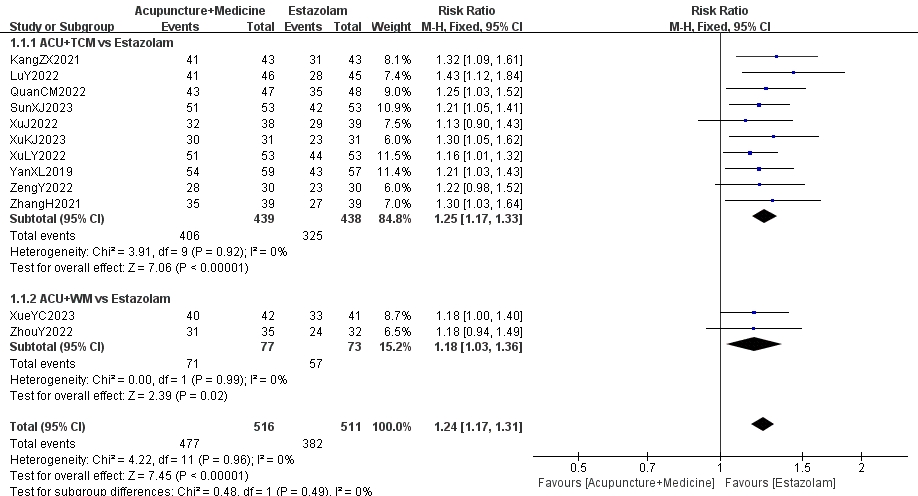

Supplement: Supplementary file 1 [file Data_Sheet_1.ZIP › Subgroup analysis/Efficiency-Subgroup analysis.jpg]

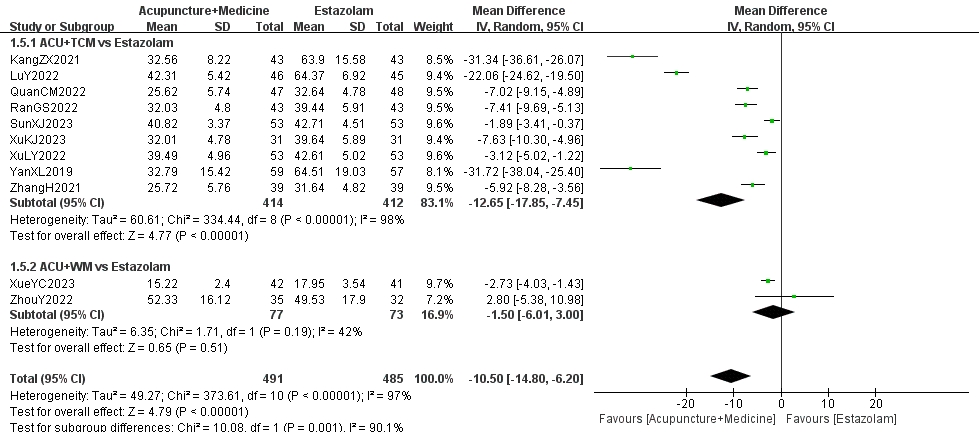

Supplement: Supplementary file 1 [file Data_Sheet_1.ZIP › Subgroup analysis/FSH-Subgroup analysis.jpg]

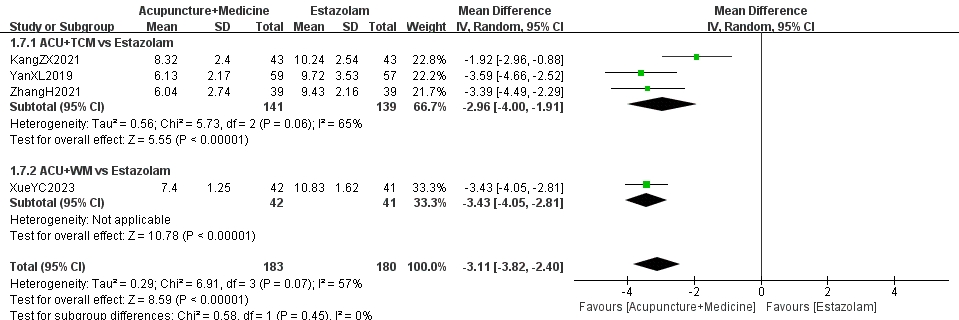

Supplement: Supplementary file 1 [file Data_Sheet_1.ZIP › Subgroup analysis/HAMA-Subgroup analysis.jpg]

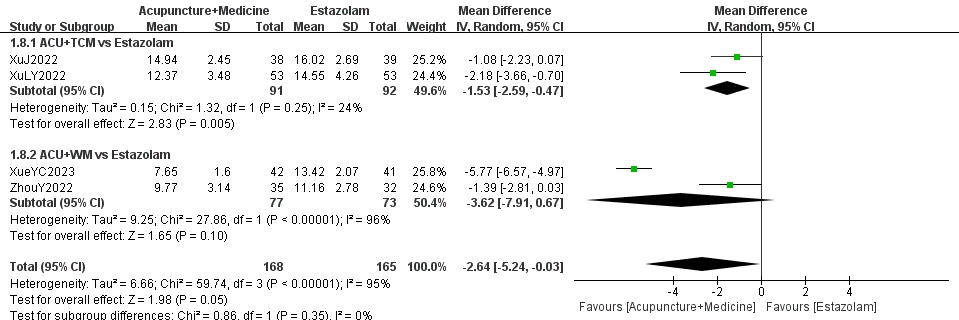

Supplement: Supplementary file 1 [file Data_Sheet_1.ZIP › Subgroup analysis/KMI-Subgroup analysis.jpg]

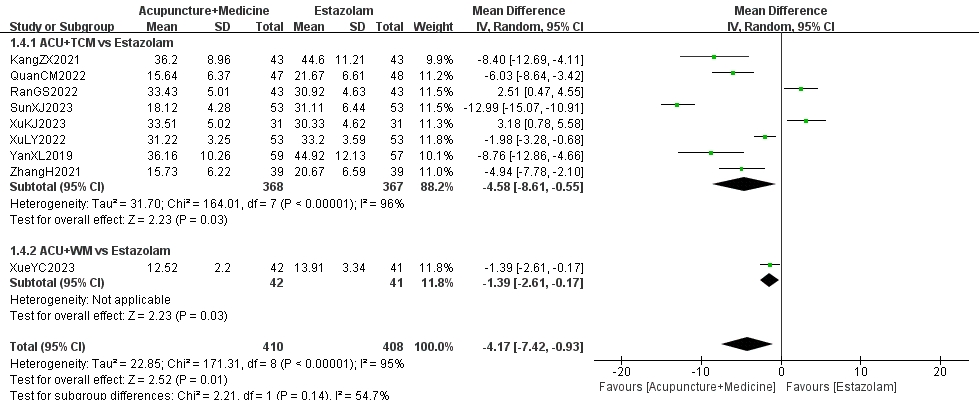

Supplement: Supplementary file 1 [file Data_Sheet_1.ZIP › Subgroup analysis/LH-Subgroup analysis.jpg]

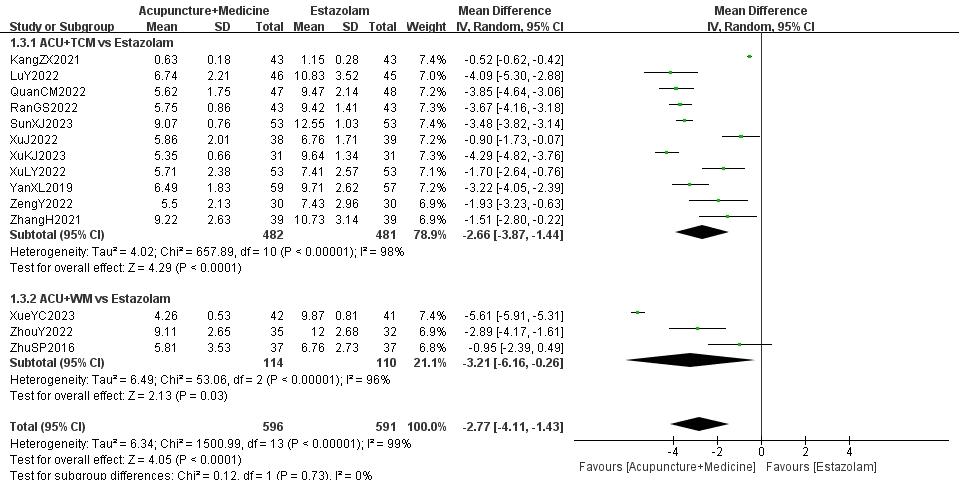

Supplement: Supplementary file 1 [file Data_Sheet_1.ZIP › Subgroup analysis/PSQI-Subgroup analysis.jpg]

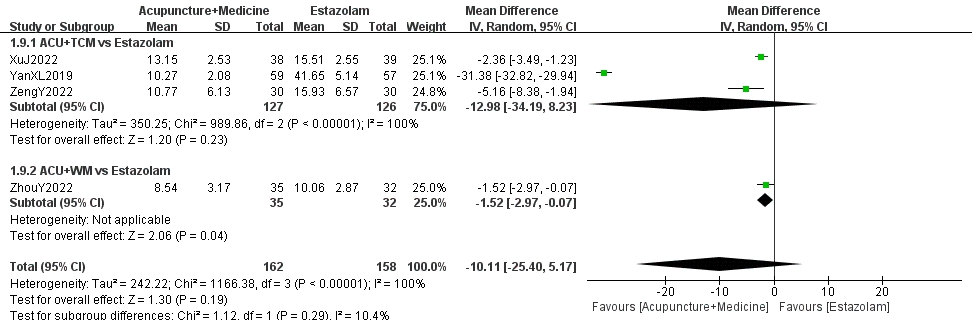

Supplement: Supplementary file 1 [file Data_Sheet_1.ZIP › Subgroup analysis/TCMS-Subgroup analysis.jpg]
